# Bio-Guided Isolation of New Compounds from *Baccharis* spp. as Antifungal against *Botrytis cinerea*

**DOI:** 10.3390/metabo12121292

**Published:** 2022-12-19

**Authors:** Ana A. Pinto, Antonio Ruano-González, Abdellah Ezzanad, Cristina Pinedo-Rivilla, Rosario Sánchez-Maestre, Juan Manuel Amaro-Luis

**Affiliations:** 1 Departamento de Química Orgánica, Facultad de Ciencias, Universidad de Cádiz, Campus Rio San Pedro, 11510 Puerto Real, Spain; 2 Laboratorio de Productos Naturales, Departamento de Química, Facultad de Ciencias, Universidad de Los Andes (ULA), Mérida C.P. 5101, Venezuela

**Keywords:** *Baccharis*, *Botrytis cinerea*, antifungal

## Abstract

*Baccharis* genus *Asteraceae* is widely used in traditional treatment against fever, headache, hepatobiliary disorders, skin ulcers, diabetes, and rheumatism, as well as an antispasmodic and diuretic. Its phytochemistry mainly shows the presence of flavonoids and terpenoids such as monoterpenes, sesquiterpenes, diterpenes, and triterpenes. Some of them have been evaluated for biological activities presenting allelopathic, antimicrobial, cytotoxic, and anti-inflammatory properties. In this paper, our research group reported the isolation, characterization, and antifungal evaluation of several molecules isolated from the dichloromethane extract from *Baccharis prunifolia*, *Baccharis trinervis*, and *Baccharis zumbadorensis* against the phytopathogen fungus *Botrytis cinerea*. The isolated compounds have not previously been tested against *Botrytis*, revealing an important source of antifungals in the genus *Baccharis*. Six known flavones were isolated from *B. prunifolia*. The dichloromethane extracts of *B. trinervis* and *B. zumbadorensis* were subjected to a bio-guided isolation, obtaining three known flavones, an α-hydroxidihydrochalcone mixture, one labdane, one triterpene, and two norbisabolenes from the most active fractions. The compounds 4′-methoxy-α-hydroxydihydrochalcone (**7A**), 3β,15-dihydroxylabdan-7-en-17-al (**8**), and 13-nor-11,12-dihydroxybisabol-2-enone (**11**) are novel. The most active compounds were the Salvigenin (**5**) and 1,2-dihydrosenedigital-2-one (**10**) with an IC50 of 13.5 and 3.1 μg/mL, respectively.

## 1. Introduction

The *Asteraceae* constitutes a defined family among flowering plants distributed primarily in the tropical areas of South America [1]. It has approximately 1500 genera and 25,000 species [2]. The genus *Baccharis* is represented by more than 500 species which are distributed mainly in Brazil, Argentina, Colombia, Chile, and Mexico [3,4]. Species of the genus *Baccharis* have been widely investigated for their pharmacological properties, well known by Indigenous populations. Medicinal properties include antidiabetic and anti-inflammatory attributes. They are also used to treat liver disease, rheumatism, digestive, hepatic, and renal disorders [3]. Phytochemically, the *Baccharis* genus produces compounds that have been identified and their biological activities studied [5]. The compounds included in *Baccharis* are mainly flavonoids [6,7] and terpenoids [8], such as diterpenes [9,10] with clerodane, labdane, and kaurane skeletons. Phenolic compounds and essential oils have been reported in the last years as important sources of natural products with interesting biological activities [11,12].

*Baccharis trinervis*, *B. prunifolia*, and *B. zumbadorensis* are widely distributed from Mexico to Argentina [13,14]. These species are used in the treatment of high fevers, edema, ulcers, and vascular cramps [5]. They are also applied in cases of dizziness, gastrointestinal disorders, and against snake venom [15]. Their different parts have been studied [16,17,18], identifying flavonoids [19] and terpenes [16,20]. Plants used in this work had been collected in Venezuela [21], and it is the first study of the antifungal activity of this species of *Baccharis* against phytopathogenic fungi. The antifungal activity of this genus has been reported mainly against human fungal infections as a part of its medicinal properties [5,13,22,23,24]. Nevertheless, the activity against phytopathogen fungi of the genus *Baccharis* has been poorly documented [25]. The traditional treatment against phytopathogen fungi is the use of chemicals that have some serious restrictions due to the effects on the natural environment. Therefore, the rational control of the diseases that they produce, is one of the biggest challenges facing the agricultural-food industry, which raises the need to discover new antifungal products of plant origin that are friendly to the environment. In this context, *Baccharis trimera* and *Baccharis ochracea* essential oils inhibited 100% of the growth of *Alternaria alternata* [26]; essential oil from *Baccharis dracunculifolia* was tested against *Fusarium graminearum* [27]; essential oils of plants, including some species of *Baccharis*, were tested with good results against *Monilinia fructicola* [28] and *Stemphylium solani* [29]; and root extracts from *Baccharis salicina* decreased the percentage of germination of uredospores of *Hemileia vastatrix* [30].

*Botrytis cinerea* is one of the most invasive and most important phytopathogen fungi in terms of economic losses [31,32,33]. It is the causal agent of the grey mold in grapes, one of the most important fruit crops worldwide. Among the possibilities of alternative control is the use of essential oils of *Baccharis trimera* and *Baccharis dracunculifolia* tested against *B. cinerea* and *Colletotrichum acutatum* that showed effectiveness as preventive and curative treatment [25].

The present work reports the bio-guided isolation of metabolites by their antifungal activity against *Botrytis cinerea* from the dichloromethane and methanol extract of *B. trinervis*, *B. prunifolia*, and *B. zumbadorensis*. The purification of the active fractions yielded six known flavones (**1**–**6**) from *B. prunifolia*; seven compounds: three flavones (**3**–**5**), an α-hydroxydihydrochalcone mixture (**7A** and **7B**), one labdane (**8**), and one triterpene (**9**) from *B. trinervis*. The compounds 4′-methoxy-α-hydroxydihydrochalcone (**7A**) and 3β,15-dihydroxylabdan-7-en-17-al (**8**) are described here for the first time. In addition, *B. zumbadorensis* yielded four compounds: two flavones (**3** and **4**) and two norbisabolenes (**10** and **11**), the 13-nor-11,12-dihydroxybisabol-2-enone (**11**) is reported for the first time. All these compounds were isolated and identified by analytical approaches (HPLC, NMR, IR, GC-MS). 

## 2. Materials and Methods

### 2.1. General Procedure

Solvents and reagents were purchased from Sigma-Aldrich, Merck, and EurisoTop^®^ brands with an analytical grade. Silica gel 60 (63–200 μm; 70–230 mesh) from Merck and columns Sephadex LH-20 from Sigma were used for column chromatography. 

HPLC purification was performed with an Elite LaChrom-Hitachi HPLC system equipped with L-2400 UV-Vis detector and L-2490 differential refractor detector. 

LiChrospher RP-18 (10 μm, 10 × 250 mm) column chromatography was used for secondary metabolites separation. The eluents were methanol, acetonitrile (Carlo Ebra^®^ reagents), and water. 

NMR spectroscopic experiments were performed on the Agilent 400 and 500 MHz spectrometers (at 25 °C). Chloroform-d (7.25 or 77.00 ppm), Acetone-d_6_ (2.05 or 29.90 ppm), or dimethylsulfoxide-d_6_ (2.50 or 39.50 ppm) (from EurisoTop^®^ brand) were used to reference the chemical shift. IR spectra were recorded on a Perkin Elmer (Spectrum BX) spectrophotometer in KBr cells. 

High-resolution mass spectra (HRESIMS) data were recorded in positive and negative modes using Waters SYNAPT equipment and MassLynx 4.1 software. 

The Optical activities were measured with a Perkin-Elmer 241 polarimeter equipped with a sodium lamp (λ = 589 nm) with chloroform as solvent at 25 °C.

ECD spectrum was measured on a JASCO J-810 spectropolarimeter at ambient temperature. The ECD curves were simulated using SpecDis 1.51 software [34].

### 2.2. Isolates and Cultures

*Baccharis trinervis* Pers was collected in July 2012 by the Arenal near Tabay road edges at an altitude of approximately 1500 m above sea level, Libertador Municipality. *Baccharis prunifolia* Steyerm was collected in July 2008 in Gavidea, located on the outskirts of Rangel Municipality, at an altitude of 2950 m above sea level. *Baccharis zumbadorensis* Badillo was collected in December 2011 in the Paramo de San José de Acequias, located on the outskirts of Campo Elias Municipality, at an altitude of 3300 m above sea level. A Voucher Specimen of each species (J.M. Amaro, No. 2366, No. 2357, and No. 2349, respectively) was deposited in the Herbarium MERF of the Faculty of Pharmacy-ULA. All the municipalities are in Merida State, Venezuela.

The culture of *B. cinerea* employed in this work, *B. cinerea* UCA 992, was obtained from grapes from the Domecq vineyard, Jerez de la Frontera, Cádiz, Spain. This culture of *B. cinerea* is deposited in the Universidad de Cadiz, Facultad de Ciencias, Mycological Herbarium Collection (UCA). The fungus was grown in an agar-tomato plate to increase the sporulation process and incubated at 25 °C the time needed for the fungus to grow and to produce spores (15 to 20 days).

### 2.3. Extraction and Bio-Guided Isolation

The leaves of the plants were recollected and dried at room temperature. The whole leaves (780 g, 2980 g, and 1750 g for *B. trinervis*, *B. prunifolia*, and *B. zumbadorensis*, respectively) were extracted with dichloromethane at room temperature for 24 h. Next, they were dried again under a hood and then ground. The ground material was extracted in a Soxhlet extractor with methanol as a solvent. The solutions from both extractions were filtered and then concentrated on a rotary evaporator at a temperature not exceeding 40 °C. The extracts obtained were kept at −4 °C. 

Dichloromethane extracts were chromatographed by Column Chromatography (CC) over silica gel, using as eluent increasing polarities of hexane–dichloromethane, and finally, methanol to yield the fractions: 

For *Baccharis prunifolia*, fourteen fractions; A (40.2 g), B (22.5 g), C (3.5 g), D (10.6 g), E (8.6 g), F (8.9 g), G (12.1 g), H (16.3 g), I (12.6 g), J (16.5 g), K (22.3 g), L (16.6 g), M (11.9 g), and N (8.7 g). For *Baccharis trinervis*, eleven fractions: A (15.2 g), B (8.6 g), C (11.2 g), D (14.7 g), E (8.5 g), F (10.6 g), G (9.0 g), H (8.6 g), I (7.7 g), J (9.5 g), and K (17.5 g). For *Baccharis zumbadorensisa*, eleven fractions: A (15.2 g), B (8.6 g), C (11.2 g), D (8.0 g), E (24.1 g), F (16.6 g), G (9.0 g), H (1.5 g), I (0.8 g), J (1.2 g), and K (0.8 g). 

**Figure 1:**
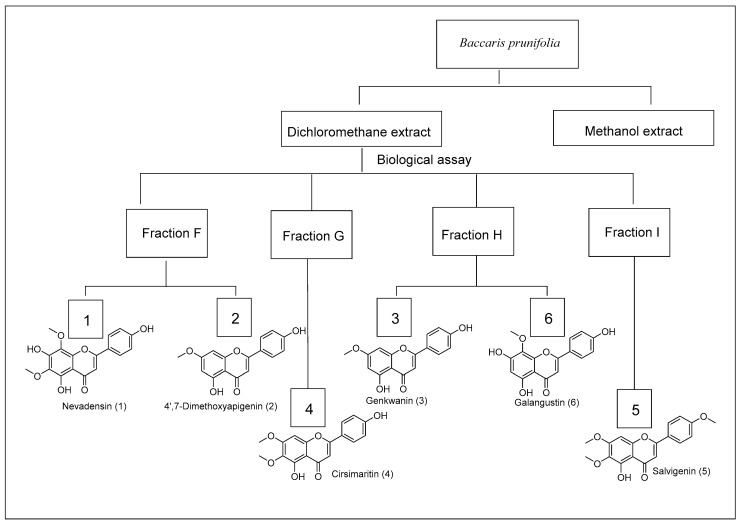
Scheme of the bio-guided isolated compounds from *Baccharis prunifolia*.

**Figure 2:**
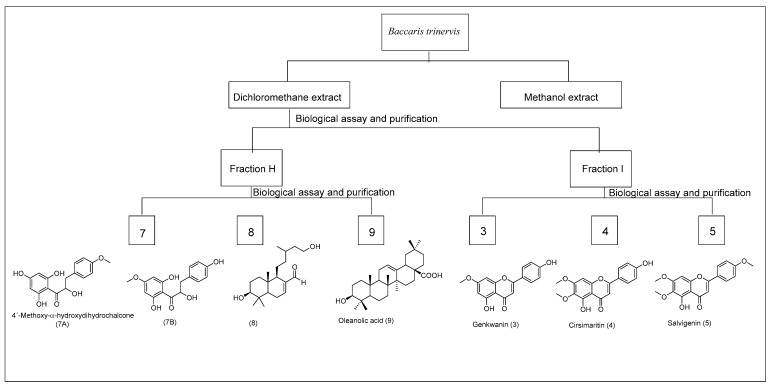
Scheme of the bio-guides isolated compounds from *Baccharis trinervis*.

**Figure 3:**
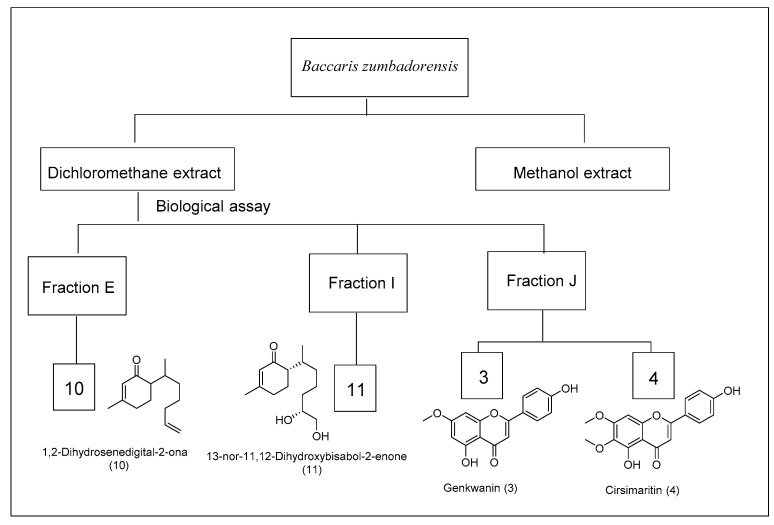
Scheme of the bio-guided isolated compounds from *Baccharis Zumbadorensis*.

The total fractions obtained from the CC of the crude extract from *Baccharis* species were tested against *B. cinerea* UCA992 by Microplate resazurin assay (Figure 1, Figure 2, Figure 3 and Figures S1–S3 [35]. 

The most active fractions were submitted to a deeper analysis as follows:

The pooled fraction F (15 g) from *B. prunifolia* was chromatographed on Sephadex LH-20 with hexane–dichloromethane–methanol mixtures 2:1:1 obtaining 15 fractions. A pure, yellow solid compound 1 was obtained in fraction F6 (0.050 g), and a pure compound 2 was obtained in fraction F9 (0.038 g). In addition, fraction H was chromatographed on preparative TLC with hexane–ethyl acetate mixtures 3:2 to obtain a pure compound characterized as Genkwanin (**3**) (0.019 g) and Galangustin (**6**) (0.010 g), both as solids. Compound **4** (0.015 g) was obtained as a solid in fraction G (2.37 g). Finally, the fraction I (2.37 g) was chromatographed on preparative TLC with hexane–ethyl acetate mixtures 3:2 to obtain a solid characterized as **5** (0.052 g).

The fraction H from *B. trinervis* (8.615 g) was submitted to CC on silica gel with hexane–acetate mixtures in increasing polarity, obtaining 15 fractions. The H5 was permeated on Sephadex LH 20 with hexane:dichloromethane:methanol 3:2:1 to obtain 1.98 mg of a mixture of 4′-methoxy-α-hydrochalcone (**7A**) and Lyonogenin (**7B**) in a 3:2 proportion. The H13 fraction (the most active against *B. cinerea*) was submitted to a CC obtaining a semi-pure compound that was purified through HPLC reverse phase chromatography. The pure compound obtained was characterized as diterpene 3β, 15-Didroxylabdan-7-en-17-al (**8**) (3.25 mg). The tri-terpene Oleanolic Acid (**9**) (1.95 mg) was isolated from fraction H5. Cirsimaritin (0.015 g) was characterized from fraction G (2.37 g). Compound 5 was isolated from I fraction (2.37 g) by chromatographic purification on preparative TLC with hexane–ethyl acetate mixtures 3:2 (0.052 g).

**2-Hydroxy-3-(4-methoxyphenyl)-1-(2,4,6-trihydroxyphenyl)propan-1-one. 4′-Methoxy-α-hydroxydihydrochalcone (7A).** Amorphous white powder. (−)-HRESIMS m/z 303.0844 [M – H]^−^ (calcd for C_16_H_15_O_5_, 303.0869), exact mass calcd for C_16_H_16_O_6_, 304.0947. ^1^H-NMR (500 MHz, CDCl_3_); δ 2.81 (m, 1H, CH_2_), 3.11 (m, 1H, CH_2_), 3.84 (s, 3H, OCH_3_), 5.37 (m, 1H, CH), 5.98 (s, 1H, CH), 5.99 (s, 1H, CH), 6.96 (d, *J* = 8.8, 1H, CH), 6.96 (d, *J* = 8.8, 1H, CH), 7.38 (d, *J* = 8.8, 1H, CH), 7.38 (d, *J* = 8.8, 1H, CH), 12.05 (s, 1H, OH).^13^C-NMR (125 MHz, CDCl_3_); δ 43.14 (CH_2_), 55.37 (OCH_3_), 79.02 (CH), 95.32 (CH), 96.65 (CH), 103.26 (C), 114.23 (CH), 114.23 (CH), 127.73 (CH), 127.73 (CH), 130.27 (C), 160.07 (C), 163.25 (C), 164.13 (C), 164.36 (C), 196.01 (CO). 

1-((1*R*,6*S*,8a*S*)-6-Hydroxy-1-(5-hydroxy-3-methylpentyl)-5,5,8a-trimethyl-1,4,4a,5,6,7,8,8a-octahydronaphthalen-2-yl)ethan-1-one or 3β,15-Dihydroxylabdan-7-en-17-al (8). (+)-HRESIMS m/z 323.2559 [M + H]^+^ (calcd for C_20_H_35_O_3_^+^, 323.2586. IR ν max; 3368 (O-H), 2930 (=C-H), 2868 (C-H), 1682 (C=O) cm^−1^. ^1^H-NMR (500 MHz, CDCl_3_); δ 0.80 (s, 3H, CH_3_), 0.91 (s, 3H, CH_3_), 0.91 (d, *J* = 7.9, 3H, CH_3_), 1.01 (s, 3H, CH_3_), 1.20 (m, 3H, CH), 1.48 (m, 2H, CH), 1.52 (m, 1H, CH), 1.65 (m, 5H, CH), 1.95 (m, 2H, CH), 2.35 (m, 2H, CH_2_), 3.27 (dd, *J*_1_ = 3.93, *J*_2_ = 11.2 1H, CH), 3.67 (m, 2H, CH_2_), 6.79 (t, *J* = 2.5, 1H, CH), 9.39 (s, 1H, CHO). ^13^C-NMR (125 MHz, CDCl_3_); δ 14.3 (CH_3_), 15.2 (CH_3_), 19.6 (CH_3_), 23.9 (CH_2_), 24.9 (CH_2_), 27.2 (CH_2_), 27.9 (CH_3_), 30.3 (CH), 36.6 (C), 37.1 (CH_2_), 38.5 (CH_2_), 38.5 (CH_2_), 38.6 (C), 50.4 (CH), 61.3 (CH_2_), 78.8 (C-OH), 144.4 (C), 151.7 (C), 194.6 (CHO).

From *B. zumbadorensis*, fraction E (24.114 g) was chromatographed on silica gel column with hexane-acetate mixtures in increasing polarity obtaining 15 fractions. Fraction E5 was submitted to CC on silica gel using hexane:dichloromethane mixtures, yielding 87 mg of flavonoid **10**. Fraction I (24.234 g) was chromatographed on silica gel CC with hexane–acetate mixtures in increasing polarity, obtaining 15 fractions. The I12 fraction was submitted to a CC obtaining a pure compound 13-nor-11,12-dihydroxybisabol-2-enone (**11**) (60 mg).

**(6*R*)-6-((6*R*)-6,7-Dihydroxyheptan-2-yl)-3-methylcyclohex-2-en-1-one (11).** (+)-HRESIMS m/z 263.1611 [M + Na]^+^ (calcd for C_14_H_24_O_3_Na, 263.1623. IR νmax; 3394 (OH), 2932 (C-H), 1656 (C=O), 909 (C=CH_2_) cm^−1^. ^1^H-NMR (500 MHz, CDCl_3_); δ 0.80 (d, *J* = 6.4, H14, 3H, CH_3_), 1.30 (m, H8, 2H, CH_2_), 1.31 (m, H9a, 1H, CH_2_), 1.44 (m, H10, 2H, CH_2_), 1.49 (m, H9b, 1H, CH_2_), 1.78 (m, H5a, 1H, CH_2_), 1.92 (m, H5b, 1H, CH_2_), 1.93 (s, H15, 3H, CH_3_), 2.12 (m, H6, 1H, CH), 2.29 (m, H4, 2H, CH_2_), 2.30 (m, H7, 1H, CH), 3.43 (m, H12, 1H, CH_2_), 3.64 (m, H12, 1H, CH_2_), 3.70 (m, H11, 1H, CH), 5.85 (s, H2, 1H, CH). ^13^C-NMR (125 MHz, CDCl_3_); δ 15.8 (C14, CH_3_), 22.6 (C5, CH_2_), 23.4 (C9, CH_2_), 24.1 (C15, CH_3_), 30.4 (C4, CH_2_), 30.7 (C7, CH), 33.2 (C10, CH_2_), 34.5 (C8, CH_2_), 49.8 (C6, CH), 66.8 (C12, CH_2_OH), 72.2 (C11, CHOH), 127.0 (C2, CH), 161.4 (C3, C), 201.3 (C1, C=O). 

### 2.4. In Vitro Antifungal Assay

The fungicidal activity of the extracts and pure compounds was tested in vitro against the plant pathogenic fungus *Botrytis cinerea* UCA992 using different methodologies according to the characteristics of the samples [36]. The estimates of the IC50 values and confidence ranges (95%, 0.0 < 0.05) of each compound were obtained from logarithmic curves by adjusting to a dose–response type curve, as implemented in the program PRISM © statistical analysis (version 5.01).

$$\%\;\mathrm{inhibition}=100-\frac{\left(\mathrm{Positive control well absorbance}\right)}{\left(\mathrm{Negative control well absorbance}\right)}\times 100$$

#### 2.4.1. Preparation of Pure Compounds Stock and Broth Microdilution Method Bioassay

The fungicidal activity of the target compounds was tested in vitro against a plant pathogenic fungi *Botrytis cinerea* UCA992. According to previous reports [37,38], ELISA equipment was employed to measure inhibition in the microplate. All materials were carefully sterilized. The tested compounds were dissolved in dimethyl sulfoxide (DMSO) at 12.5 mg/mL as stock solution. From this solution, a work-solution was prepared, dissolving 13 μL in 1300 μL of Sabourad-glucose broth. Firstly, 100 μL of Sabourad-medium were added to the second and subsequent columns, then 200 μL of work-solution were added to the six first rows of the first column in the plate. Dilutions were prepared taking 100 μL from the first column and mixed to homogeneity with the second one, and this process was repeated for all the columns obtaining a concentration gradient. Next, 100 μL aliquots of a spore solution (5 × 10^4^ spores/mL) of the strain *B. cinera* UCA992 were inoculated in the first three rows of microplates with Sabourad-glucose liquid medium and the corresponding compound in a typical concentration range from 62.50 ppm to 0.061 ppm (concentration range from the first column to the last one), therefore reaching each microplate a total volume of 200 μL. Then, the plate was incubated for 72 h at 28 °C with a fungal control plate (all the microplates with 100 μL of a spore solution (5 × 10^4^) and 100 μL of Sabourad-medium), to compare with the normal fungal growth and a medium control plate (all the microplates with 100 μL of sterilized water and 100 μL of Sabourad-medium) to eliminate the absorbance relative to the medium. Once the incubation time was completed, the absorbance of the three kinds of plates was measured, and 10 μL of a 0.027 M resazurin solution was added to all the microplates to detect contamination [39,40]. This process was performed at least three times for each compound to gather a statistical data and analyzed by Prism^®^ to determinate the IC50 value. 

For data treatment, we perform a minimum of 9 experiments. It usually takes us three days to complete a study, performing three repetitions per day. Each experiment is submitted to the Grubbs test and then is represented graphically, and an equation associated with the data used to obtain a first approximation of the IC50 value is obtained. Once all the graphs and IC50 values of the “*n*” experiments are obtained, we discard the extreme values and keep the central values (the volume of “n” data will present a Gaussian curve). The estimates of the IC50 values and confidence ranges (95%, 0.0 < 0.05) of each compound were obtained from logarithmic curves by adjusting to a dose–response type curve, as implemented in the program PRISM © statistical analysis (version 5.01).

#### 2.4.2. Bio-Guided Antifungal Assays: Use of Resazurin as Inhibition Indicator

**Figure 4:**
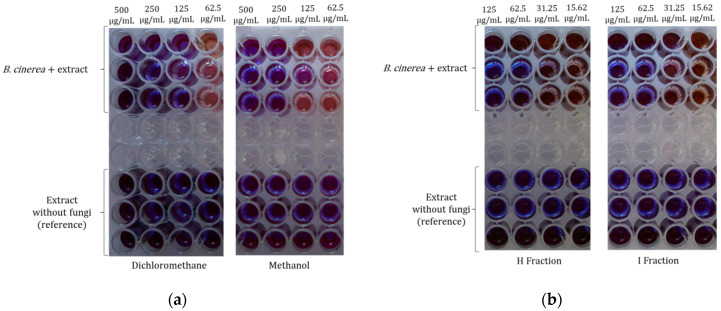
Microplate analysis of resazurin data from extracts and fractions against *Botrytis cinerea*: (**a**) dichloromethane and methanol extracts at gradient concentration keep the inhibition at 125 ppm and 250 ppm, respectively; (**b**) fractions H and I from *B. trinervis* at gradient concentration keep the inhibition at 62.5 ppm.

The minimal inhibitory concentration for microorganism growth (MIC) was determined in triplicate by using the microdilution broth method in 96-well microplates. Samples were dissolved in DMSO at 12.5 mg/mL obtaining a solution of 500 ppm (take 40 μL of stock solution and 960 μL of the medium solution Sabourad-glucose in an Eppendorf). A total of 100 μL of this solution was added into the first well with 100 μL of water or solution of the spores from *B. cinerea* UCA992 obtaining a 250 ppm concentration. The final DMSO concentration should be less than 2% to not interfere with the assay. Concentrations ranging from 250 to 0.05 ppm were achieved. One inoculated well was included, to allow for control of the adequacy of the broth for organism growth. One non-inoculated well, free of antimicrobial agent, was also employed, to ensure medium sterility. Irgasan was used as positive control. The microplates (96-wells) were incubated at 28 °C for 72 h. After the incubation time, 10 μL of a solution of resazurin (270 mg in 40 mL of distilled and sterilized water) was added to all the microplates to indicate microorganism viability and check non-inoculated well was free of contamination, and then microplates were sealed with a sterile adhesive polyester film (50 μm; VWR^®^ Microplate Sealing Film) and incubated (28 °C with artificial light) for 24 h more. The MIC values of extract from *Baccharis* spp. were determined as the lowest concentration in which the resazurin (purple) did not bio-transform to resorufine (red/brown) (see Figure 4) due to the inhibition of *B. cinerea* growth. 

#### 2.4.3. Poisoned Food Medium Assay

The fungicidal properties of most active compounds, **5** and **10**, were assessed by the “poisoned food” technique (Figures S28 and S29) [41]. The bioassay was carried out by measuring radial growth inhibition on an agar medium in a Petri dish in the presence of test compounds at 28 °C. The test compound was dissolved in ethanol, resulting in a final compound concentration of 0.06–30 µg/mL. The final ethanol concentration was identical in the control and treated cultures. The medium was poured into 9 cm diameter sterile Petri dishes, and a 5 mm diameter mycelial disk of *B. cinerea* cut from an actively growing culture (two days of growth) was placed in the center of the agar plate. Radial growth was measured for three days. Three independent experiments and three replicates per treatment were conducted. The fungicide irgasan was used as a standard for comparison in this test. 

#### 2.4.4. Statistical Analysis

The data were analyzed using an ANOVA test with PRISM © statistical analysis software (version 5.01). Dose–response analysis was performed to estimate the IC50 values with 95% confidence ranges.

## 3. Results

### Bio-Guided Isolation and Identification of New Compounds

The first part of this study was to test the antifungal activity of the DCM and methanol extracts from the aerial parts of *Baccharis* species (Table 1). The antifungal study was performed using the microdilution method [42].

**Figure 5:**
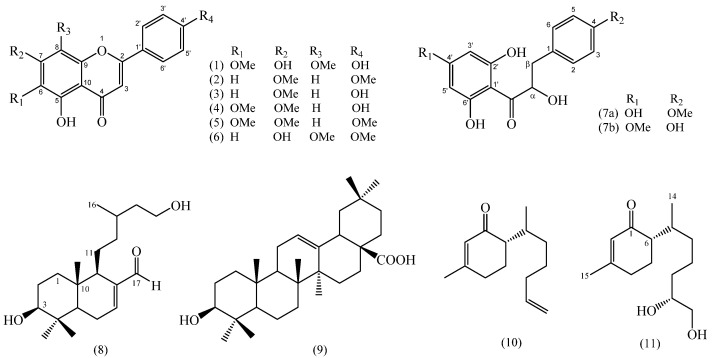
Structures of compounds isolated from *Baccharis* species (*B. trinervis*, *B. prunifolia*, and *B. zumbadorensis*). New compounds isolated from *Baccharis* spp.: 4′-Methoxy-α-hydroxydihydrochalcone (**7A**), 3β,15-dihydroxylabdan-7-en-17-al (**8**), and (6*R*)-6-((6*R*)-6,7-dihydroxyheptan-2-yl)-3-methylcyclohex-2-en-1-one (**11**).

The DCM extracts gave the best results as they were fractioned, and the assays were repeated with the fractions. The chromatographic analysis by CC of the active fractions led to the isolation of nine known compounds and three novel compounds (Figure 5). Compounds from fractions F, G, H, and I (*B. prunifolia* (750 g)) were identified as Nevadensin (**1**) [43], 4′,7-dimethoxyapigenin (Sakuranetin) (**2**) [44,45], Genkwanin (**3**) [46,47], Cirsimaritin (**4**) [48], Salvigenin (**5**) [49], and Galangustin (**6**) [50]. From the *B. trinervis* extract (288 g), the fraction H yielded 4′-methoxy-α-hydroxydihydrochalcone (**7A**), Lyonogenin (**7B**) [51], and 3β,15-dihydroxylabdan-7-en-17-al (**8**); and from fraction I yielded the compounds **3**, **4**, and **5**. Finally, the extract from *B. zumbadorensis* (267 g) yielded the following compounds: 1,2-Dihydrosenedigital-2-one (**10**) [52] from fraction E, compound **11** from fraction I, and compounds **3** and **4** from fraction J.

The previously reported compounds were identified by comparing their obtained spectroscopy data with those in the literature. Nevadensin (**1**), Genkwanin (**3**), Cirsimaritin (**4**), and Salvigenin (**5**) have been reported as a constituent of the aerial parts of different species of *Baccharis* [19,43,53,54,55]. However, it is the first time they have been reported in *B. prunifolia*, presenting a phytochemical contribution to this species. They present different biological activities: hypotensive, anti-inflammatory, cytotoxic, and antimicrobial [56,57,58,59,60,61,62,63]. In addition, the compounds 4′,7-dimethoxyapigenin (**2**) and 5,7-dihydroxy-4′,8-dimethoxyflavone (Galangustin) (**6**) were described here for the first time in the genus *Baccharis*. They present high antioxidant activity [64,65,66], and cytotoxicity has been tested [45].

A compound never isolated before in the genus *Baccharis*, 13-nor-bisabol-2,11-dienone (**10**), was isolated from fraction E, extracted from *B. zumbadorensis*; the new compound 13-nor-11,12-dihydroxybisabol-2-enone (**11**) was isolated from fraction I; and the known Genkwanin (**3**) and Cirsimaritin (**4**) were isolated from fraction J. The compound 13-nor-bisabola-2,11-dienone (**10**), known by the name of 1,2-dihydrosenedigital-2-one, has only been previously reported in the species *Senecio digitalifolius* (*Asteraceae*) [67]; while 13-nor-11,12-dihydroxybisabol-2-enone (**11**) is a norsesquiterpene of the bisabolene series which has not been described previously in the literature.

The compound 4′-methoxy-α-hydroxydihydrochalcone (**7A**), isolated from the α-hydroxydihydrochalcone mixture extracted from *B. trinervis*, is described here for the first time (Figures S4–S6 and S7a,b). Compound (**7A**) was elucidated from a mixture in which the isomer Lyogenin (**7B**) was the major compound, obtained as an amorphous solid. The HRESIMS (-) data of this compound (detected m/z 303.0869 calculated for C_16_H_15_O_6_ [M-H]^−^ m/z 303.0869) resulted in the molecular formula C_16_H_16_O_6._ Comparison of the spectroscopic data with those of (**7B**) reported in the literature [47] indicates that the 4-OH and 7′-OMe groups were interchanged. The ^1^H-NMR spectrum of **7A** showed a low field AA’BB’ system integrating to two protons at δH: 7.38 (H-2A/H-6A) and δH: 6.96 (H-3A/H-5A) with a coupling constant of 8.8 Hz, typical of a para-substituted benzene ring (Table 1 and Figure S4). ^13^C-NMR spectrum of **7A** showed signals at δ_C_ 103.7 (C), 114.2 (2 × CH), 127.7 (2 × CH), 130.3 (C), 160.1 (C), 163.3 (C), 164.1 (C), and 164.4 (C) corresponding to two benzoyl groups, δ_C_ 196.0 corresponding to an α,β unsaturated carbonyl and δ_C_ 55.4 corresponding to OMe group (Table 1 and Figure S5). Correlations H6 (δH 7.38) with carbon α (δ_C_ 79.02) and H-β1 (δH 3.11) with C-1 (δC 130.27) were confirmed in ^1^H-^13^C HMBC spectrum. The correlation of the OMe group (δH 3.84) with C4 (δ_C_ 160.1) confirmed the presence of the OMe group at C4 (Figure S6a,b). According to this result, compound **7A** was assigned as 4′-methoxy-α-hydroxydihydrochalcone.

**Figure 6:**
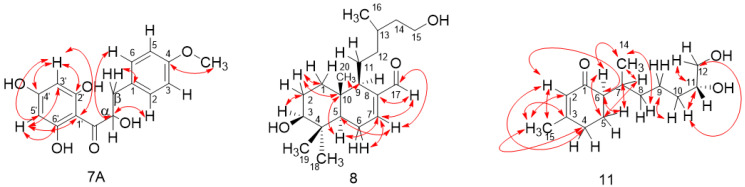
Structural analysis of **7A**, **8**, and **11**, key ^1^H-^13^C HMBC correlations.

Compound (**8**) was obtained as a colorless amorphous solid. The HRESIMS (+) data of this compound (m/z: 323.2559, calculated for C_20_H_35_O_3_ [M + H]^+^) resulted in the molecular formula C_20_H_34_O_3_, with four degrees of unsaturation_._ The ^1^H-NMR spectrum of **8** revealed the presence of two proton groups adjacent to an oxygen group (δH-3: 3.27 and δH-15: 3.67), a proton in sp^2^-carbon (*δ*H-7: 6.79), a couple of proton signals near to an alkene group (δ-H6: 2.35 and δ-H9: 1.95), four methyl groups (δ-H18: 1.01, δ-H19: 0.91, δ-H20: 0.80, and δ-H16: 0.91), and an aldehyde signal at *δ*H-17: 9.39. Methylene signals H1, H2, and H11 (δ-H1: 1.65, δ-H2: 1.65, δ-H11: 1.48) were presented as multiplets and two methylene signal were assigned as H12 and H14 (δ-H12: 1.20, 1.95, and δ-H14: 1.20, 1.65), both signals as multiplets (Table 2 and Figures S8 and S10). The ^13^C NMR spectrum presents signals at *δ*_C_: 144.4 (C-8), 151.7 (C-7), 194.6 (CHO)) confirming the presence of an aldehyde and an alkene group (Table 2 and Figure S9). The analysis of the ^1^H-^13^C HMBC spectrum showed a heterocyclic ring of 6/6 carbon connected by C5 and C10. Furthermore, the ^1^H-^13^C HMBC correlations between H-11 and C-9 (Figure 6 and Figure S11) confirm the presence of the alcohol moiety at C-9. NOESY correlations H-5/H-9, H-19/H-20, H-3/H-5, and H-3/H-18, support the proposed structure (**8**) (Figure 6 and Figure S13a). All ^1^H-^13^C HMBC correlations are shown in Figure 6. 

**Figure 7:**
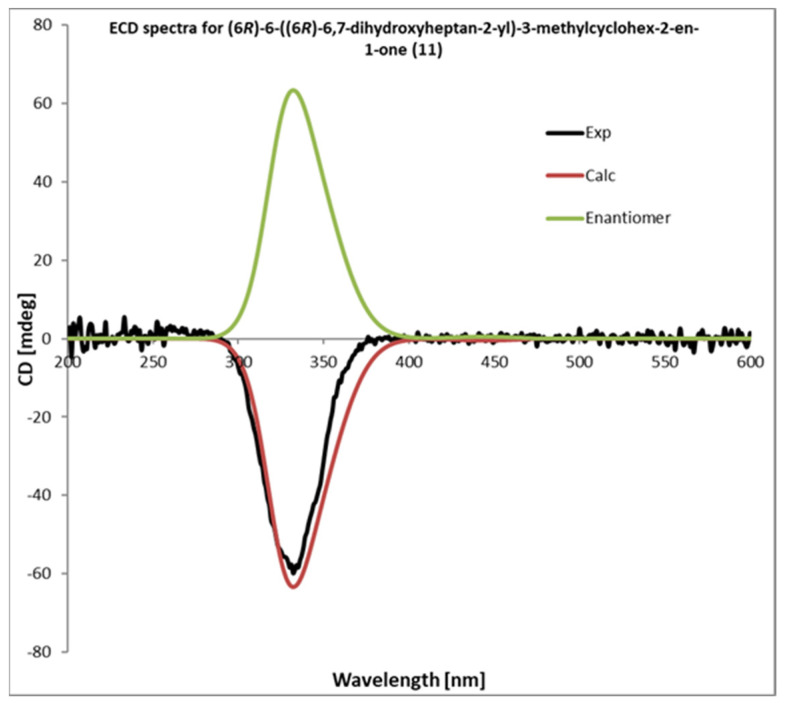
Experimental and calculated ECD spectra of compound **11** permit to assign the configuration as (6*R*)-6-((6*R*)-6,7-dihydroxyheptan-2-yl)-3-methylcyclohex-2-en-1-one.

Compound **11** was isolated as a yellow oil with a specific rotation of [α]_D_^25^: −24.4. The analysis of the high-resolution mass spectrum (HRESIMS (+) [M + Na]^+^: 263.1623, found: 263.1611) confirms the molecular formula C_14_H_24_O_3_. NMR spectroscopic data of **11** showed similarities with those published from the known compound **10 [51]**. The ^1^H-NMR spectrum (Table 2 and Figure S14) showed the presence of a methyl group (*δ*H-14: 0.80), a proton next to an alkene system (*δ*H-4: 2.29), and a methyl group on sp^2^-carbon (*δ*H-15: 1.93). Furthermore, three oxygenated groups (*δ*H-11: 3.70 and *δ*H-12: 3.64 and 3.63) and an olefinic group (*δ*H-2: 5.85) were present. The ^13^C-NMR spectrum (Table 2 and Figure S15) showed the presence of an α-β unsaturated carbonyl group *δ*C: 201.3 (C-1), an olefinic group *δ*C: 161.4 (C-3), 127.0 (C-2), and two oxygenated carbons, *δ*C: 72.2 (C-11) and 66.8 (C-12). The ^1^H-^13^C HMBC correlation observed between H-6 and C-5, H-6 and C-14, H-7 and C-5, H-7 and C-14, and between H-8 and C-14 supports the idea that the di-alcohol moiety was connected to C-6 (Figure S17). All ^1^H-^13^C HMBC correlations are shown in Figure 3. According to this result, compound **11** was assigned as 13-nor-11,12-dihydroxybisabol-2-enone. The absolute configuration for **11** was confirmed by comparing their theoretical and experimental ECD spectra. The calculated and measured ECD curves matched well, leading to the assignment of the absolute configuration of **11** as (6*R*)-6-((6*R*)-6,7-dihydroxyheptan-2-yl)-3-methylcyclohex-2-en-1-one (Figure 7).

## 4. Antifungal Assay

The pure compounds isolated and identified from the three species in this study were tested against the phytopathogenic fungus *B. cinerea* UCA992. Some of them have been described to have antimicrobial activities against some bacteria and fungi, but compounds **3**, **4**, **5**, and **8** had not previously been tested against *B. cinerea*. Salvigenin (**5**) (IC50 of 41.1 μM) isolated from *B. prunifolia* and *B. trinervis*, and 6(*R*)-6-((*R*)-hept-6-en-2-yl)-3-methylcyclohex-2-en-1-one (**10**) (IC50 15.2 μM) isolated from *B. Zumbadorensis* showed the highest activity (Figures S23 and S26). 

The flavonoids **1**, **2**, **3**, and **4** showed moderate activity. Nevadensin (**1**) had the lowest activity (IC50 of 57.0 ppm), 4′, 7-dimethoxyapigenin (**2**); Genkwanin (**3**) and Cirsimaritin (**4**) had a similar activity (IC50 of 31.3, 35.9, and 38.9 ppm); all of them have a hydroxyl group in C-5. On the other hand, Genkwanin (**3**) has a similar structure to **2** with the main difference being the free hydroxyl group presented in C-4′ (Figure 5), so this free hydroxyl group could be related to the activity increase in compound **3** (IC50 of 35.9 ppm) (see Supporting Material). 

Salvigenin (**5**) and Galangustin (**6**) showed similar activities. In both cases, there is a hydroxyl group in C-5 and a methoxyl group in C-4′ (compound **5**) and C-8 (compound **6**). Salvigenin (**5**) showed the highest activity of all flavonoid compounds tested. A structural comparison with compounds **3** and **6** shows the importance of the free hydroxyl group in C-4′ as seen in the previous case (compounds **2** and **3**) and the presence of a methoxyl group in C-7. In conclusion, for this family of compounds, the IC data confirms the importance of presenting at least a free hydroxyl group to increase the polarity, which increases the solubility in polar media (a hydroxyl group in C-5). A hydroxyl group in C-5 and a methoxyl group in C-8 (compound **6**) or C-4′ (compound **5**) could be related to the more efficient structural requirements for a high activity against *B. cinerea*. Compound **8**, a novel compound identified from *B. trinervis*, showed a lower activity with an IC50 of 70.04 ppm.

**Figure 8:**
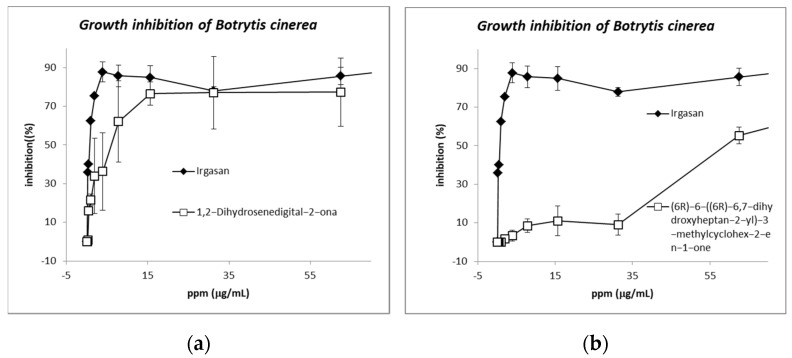
Data obtained from the antifungal test of the isolated compounds 1,2-dihydrosenedigital-2-ona (**10**) and (6*R*)-6-((6*R*)-6,7-dihydroxyheptan-2-yl)-3-methylcyclohex-2-en-1-one (**11**) from *Baccharis* sp. Comparison of the activities: (**a**) compound **10** and (**b**) compound **11**, showing completely different activities (IC50 of 3.1 ppm and 59.1 ppm, respectively).

Compounds **10** and **11** showed completely different activities (IC50 of 3.1 ppm and 59.1 ppm, respectively); this data is the first report on the fungicide activity of these structures. Compound **10**, with an alkene group in C-11-C-12 showed higher activity than the oxidized analog compound **11** (see Figure 8). This fact manifests the importance of the oxidation reaction in *B. cinerea* as a part of a detoxification pathway [68,69,70].

The most active compounds (**5** and **10**) were also tested by the poisoned food technique [36,71], obtaining similar results to the previous micro-dilution data (Figures S28 and S29).

## 5. Conclusions

Three species from the *Baccharis* genus (*Baccharis prunifolia*, *Baccharis trenervis*, and *Baccharis zumbadorensis*) were studied in order to isolate new compounds with antifungal activity against the phytopathogen fungus *B. cinerea* UCA992. This is the first report of biological assays against the phytopathogen *Botrytis cinerea* which tests these isolated compounds.

For this purpose, the extracts and the fractions from an initial chromatographic analysis were submitted to bio-guided isolation. Phytochemical investigation of the most active fractions of DCM extracts allowed for the identification of twelve compounds. Three of them reported here for the first time: (**7A**), (**8**), and (**11**). All compounds were tested against *Botrytis cinerea* UCA 992. The most active compounds were Salvigenin (**5**) with an IC50 of 13.5 ppm and 1,2-dihydrosenedigital-2-one (**10**) with an IC50 of 3.1 ppm.

## Figures and Tables

**Table 1:** MIC data of the extracts from *Baccharis* spp. against *B. cinerea* UCA992. The most active fractions were those from dichloromethane.

Species	Part of the Plant	Extract	MIC (μg/mL)
*Baccharis prunifolia*	Leaves	Dichloromethane	125
		Methanol	125
*Baccharis trinervis*	Leaves	Dichloromethane	125
		Methanol	250
*Baccharis zumbadorensis*	Leaves	Dichloromethane	125
		Methanol	250
Irgasan			0.23

**Table 2:** NMR data of novel compounds isolated from Baccharis species.

	7A			8			11	
	δ_H_ Mult(J in Hz)	δ_C_		δ_H_ Mult(J in Hz)	δ_C_		δ_H_ Mult(J in Hz)	δ_C_
1	-	130.3	1	1.65, m	27.2	1	-	201.3
2	7.38, d (8.8)	127.7	2	1.65, m	38.5	2	5.85, m	127.0
3	6.96, d (8.8)	114.2	3	3.27, dd (3.9, 11.2)	78.8	3	-	161.4
4	-	160.1	4	-	38.6	4	2.29, m	30.4
5	6.96, d (8.8)	114.2	5	1.20, m	49.0	5	a 1.78 m	22.6
							b 1.92, m	
6	7.38, d (8.8)	127.7	6	2.35, m	24.9	6	2.12, m	49.8
α	5.37, m	79.0	7	6.79, t	151.7	7	2.30, m	30.7
β	3.11, m	43.1	8	-	144.4	8	1.30, m	34.5
	2.81, m							
1′	-	103.3	9	1.95, m	50.4	9	a 1.31	23.4
							b 1.49	
2′	-	164.4	10	-	36.6	10	1.44, m	33.2
3′	5.99, s	96.7	11	1.48, m	23.9	11	3.70, m	72.2
4′	-	164.1	12	1.95, m 1.20, m	37.1	12	3.64–3.43, m	66.8
5′	5.98, s	95.3	13	1.52, m	30.3	13	-	-
6′	-	163.3	14	1.65, m 1.20, m	38.5	14	0.80, d (7.0)	15.8
C=O	-	196.0	15	3.67, m	61.3	15	1.93, s	24.1
OCH_3_	3.84, s	55.4	16	0.91, d (7.9)	19.6	-	-	
OH	12.05, s	-	17	9.39, s	194.6	-	-	
-	-	-	18	1.01, s	27.9	-	-	
-	-	-	19	0.91, s	15.2	-	-	
-	-	-	20	0.80, s	14.3	-	-	

## Data Availability

The data presented in this study are available in the main article and the supplementary materials.

## Supplementary Materials

The following supporting information can be downloaded at: https://www.mdpi.com/article/10.3390/metabo12121292/s1. Isolation procedure: SI-1: Scheme of the bio-guided isolated compounds from Baccharis prunifolia; SI-2: Scheme of the bio-guides isolated compounds from Baccharis trinervis; SI-3: Scheme of the bio-guided isolated compounds from Baccharis Zumbadorensis. NMR data: SI-4: ^1^H-NMR spectrum of compound 7A in CDCl_3_ (400 MHz); SI-5: ^13^C-NMR spectrum of compound 7A in CDCl_3_ (100 MHz); SI-6: gHMBC spectrum of compound 7A in CDCl_3_.; SI-7: gHSQC spectrum of compound 7A in CDCl_3_; SI-8: ^1^H-NMR spectrum of compound 8 in CDCl_3_ (400 MHz); SI-9: ^13^C- NMR spectrum of compound 8 in CDCl_3_ (100 MHz); SI-10: gCOSY spectrum of compound 8 in CDCl_3_ (400 MHz); SI-11: gHMBC spectrum of compound 8 in CDCl_3_; SI-12: gHSQC spectrum of compound 8 in CDCl_3_; SI-12a: gHSQC correlations of compound 8; SI-13: NOESY spectrum of compound 8 in CDCl_3_; SI-14: ^1^H-NMR spectrum of compound 11 in CDCl_3_ (400 MHz); SI-15: ^13^C- NMR spectrum of compound 11 in CDCl_3_ (100 MHz); SI-16: gCOSY spectrum of compound 11 in CDCl_3_; SI-17: gHMBC spectrum of compound 11 in CDCl_3_; SI-18: gHSQC spectrum of compound 11 in CDCl_3_; SI-18a: gHSQC correlations of compound **11**. Antifungal activity against *Botrytis cinerea* UCA 992: From SI-19 to SI-29.
