# Supplementary material for: Bio-Guided Isolation of New Compounds from Baccharis spp. as Antifungal against Botrytis cinerea
Source: Metabolites. 2022 Dec 19;12(12):1292. doi: 10.3390/metabo12121292 (PMC9781812; doi:10.3390/metabo12121292)
Supplement: Supplementary file 1 [file metabolites-12-01292-s001.zip › metabolites-2060314-supplementary.pdf]

Article

Bio-guided isolation of new compounds from *Baccharis* spp. as antifungal against *Botrytis cinerea*.

Ana A. Pinto [1,2], Antonio Ruano-González [1,*], Abdellah Ezzanad [1], Cristina Pinedo-Rivilla [1], Rosario Sánchez-Maestre [1] and Juan Manuel Amaro-Luis [2]

[1] Departamento de Química Orgánica, Facultad de Ciencias, Universidad de Cádiz, Campus Rio San Pedro, 11510 Puerto Real, Spain
[2] Laboratorio de Productos Naturales, Departamento de Química, Facultad de Ciencias, Universidad de Los Andes (ULA), Mérida C.P. 5101, Venezuela
* Correspondence: antonio.ruano@uca.es

Table of contents

Figure SI- 2: Scheme of the bio-guides isolated compounds from *Baccharis trinervis*........... 2
Figure SI- 3: Scheme of the bio-guided isolated compounds from *Baccharis Zumbadorensis*.3
Figure SI-6a: gHMBC spectrum of compound **7A**; Correlation between H-1 (3.11) and C1 (130,3) in compound **7A.** 6
Figure SI-6b: gHMBC spectrum of compound **7A**; Correlation between H2/H6 with C2/C6 and H3/H5 with C3/C5 in compounds**7A** and **7B.**........ 6
Figure SI- 7b: gHSQC correlations in **7A** and **7B**........ 8
Figure SI-13a: NOESY spectrum of compound **8**; Correlation between H19 and H20...... 12
Figure SI-18a: gHSQC correlations of compound **11**........ 16
SI- 19- Nevadensin (1). 17
SI- 20- 4',7-Dimethoxyapigenin (2)......... 18
SI-21- Genkwanin (3)......... 19
SI- 22- Cirsimaritin (4)......... 20
SI- 23- Salvigenin (5). 21
SI- 24- Galangustin (6). 22
SI- 25- 3β,15-Dihydroxylabdan-7-en-17-al (8). 23
SI- 26- 1,2-Dyhidrosenedigital-2-ona (10). 25
SI- 27- 13-nor-11,12-Dihydroxybisabol-2-enone (11). 26
SI- 29- Plate bioassay. Compound 10........ 28
SI- 30- Positive control. Microplate Bioassay (irgasan). 29
SI- 31- Positive control. Plate Bioassay (irgasan)......... 30

Isolation procedure:

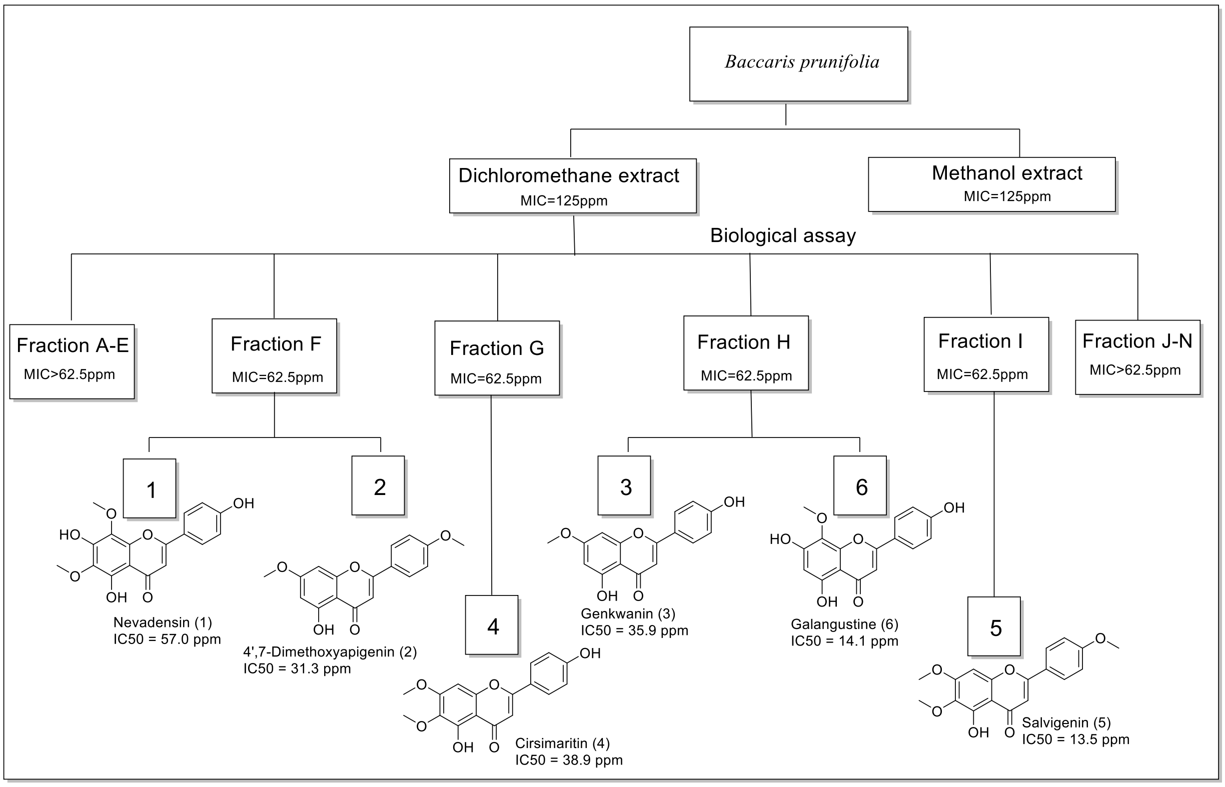

Figure SI- 1: Scheme of the bio-guided isolated compounds from *Baccharis prunifolia.*

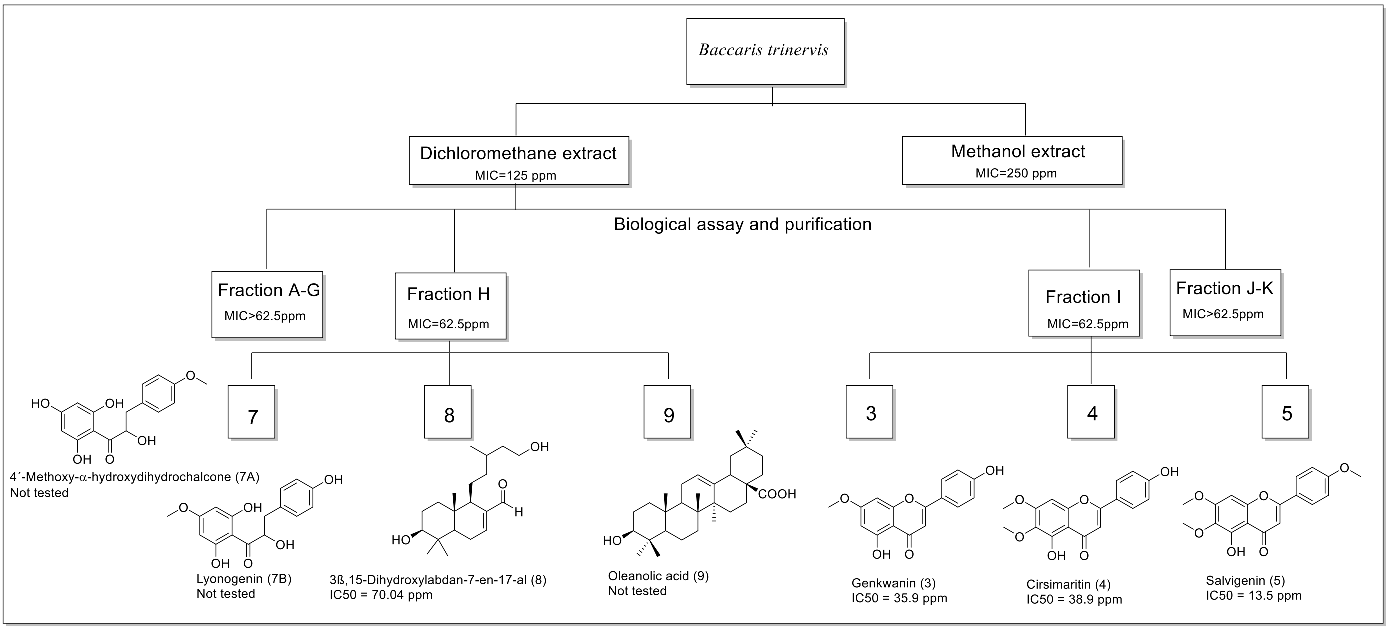

Figure SI- 2: Scheme of the bio-guides isolated compounds from *Baccharis trinervis*.

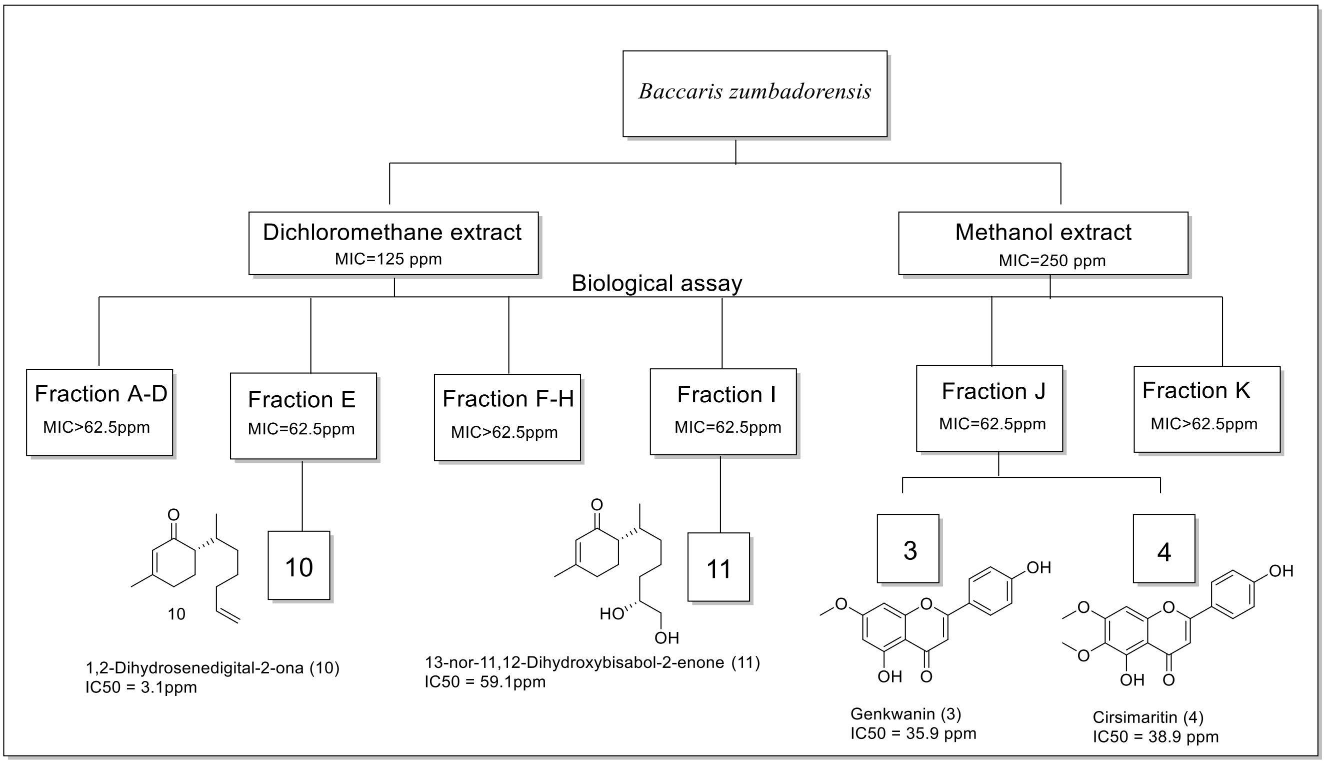

Figure SI- 3: Scheme of the bio-guided isolated compounds from *Baccharis Zumbadorensis*.

1- NMR spectroscopic data

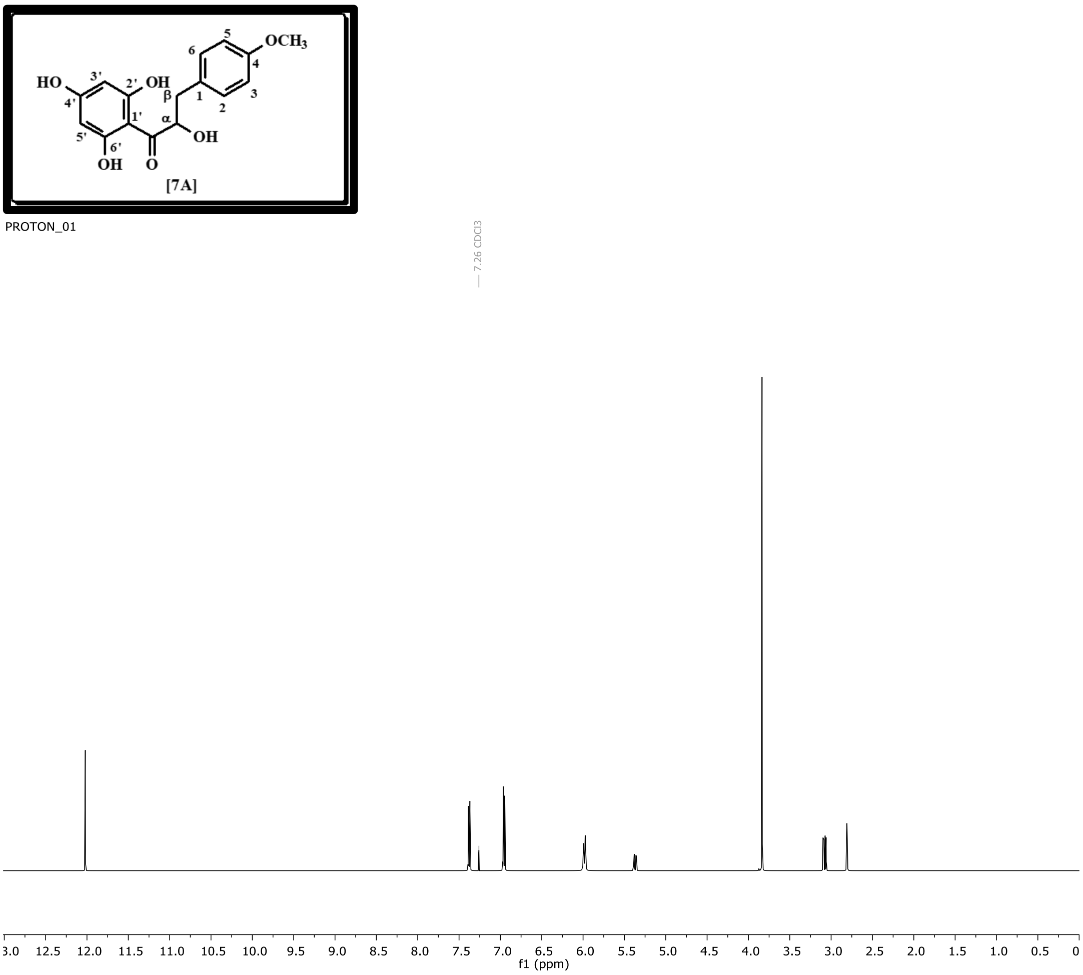

Figure SI- 4: ^{1}H-NMR spectrum of compound **7A** in $CDCl_3$ (400MHz).

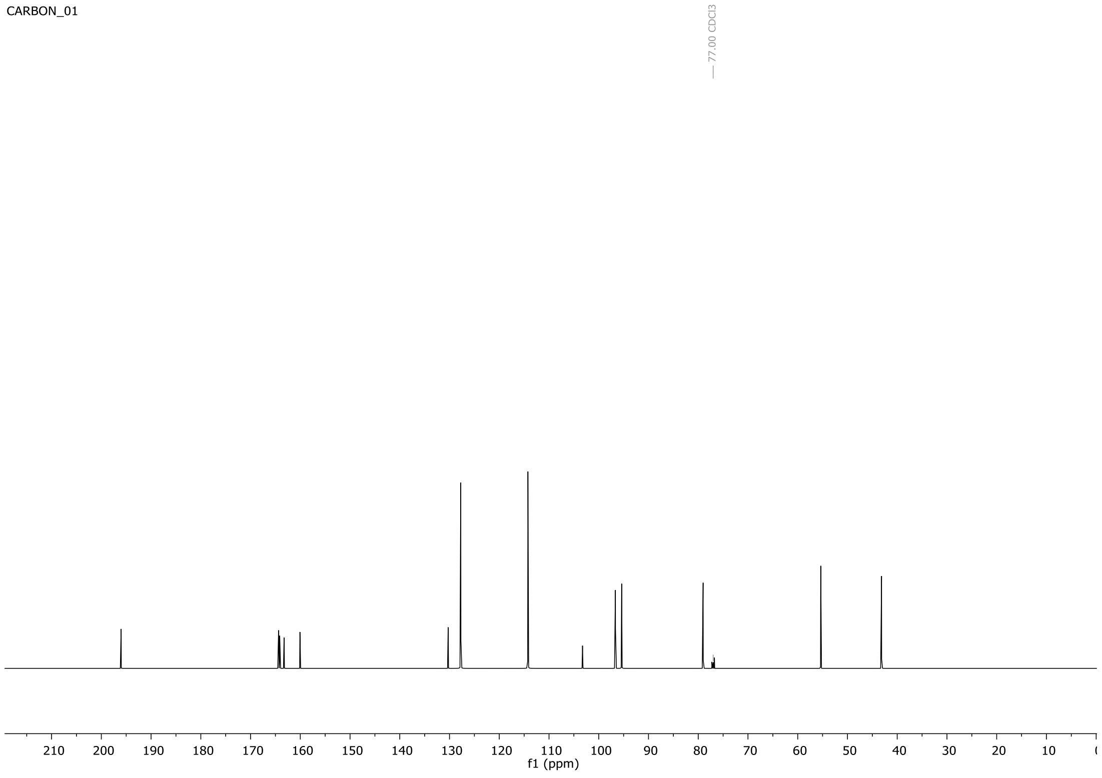

Figure SI- 5: ^{13}C-NMR spectrum of compound **7A** in $CDCl_3$ (100MHz).

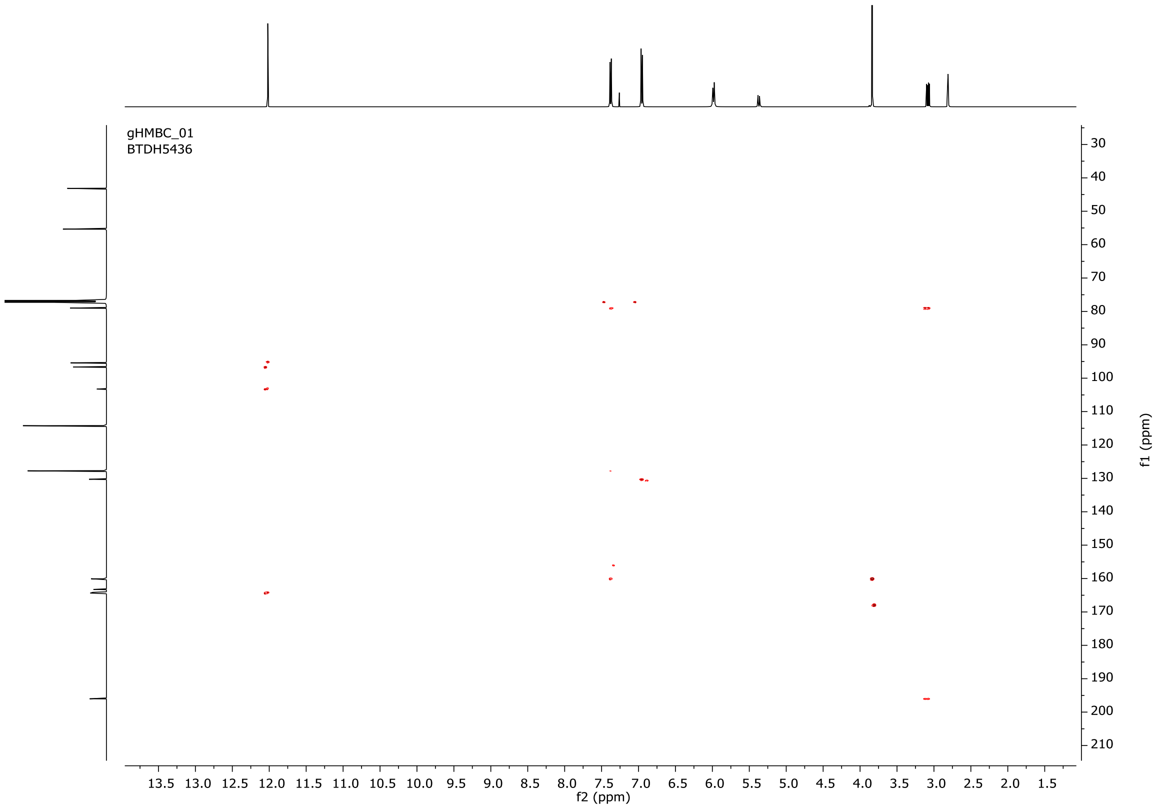

Figure SI- 6: gHMBC spectrum of compound **7A** in $CDCl_3$.

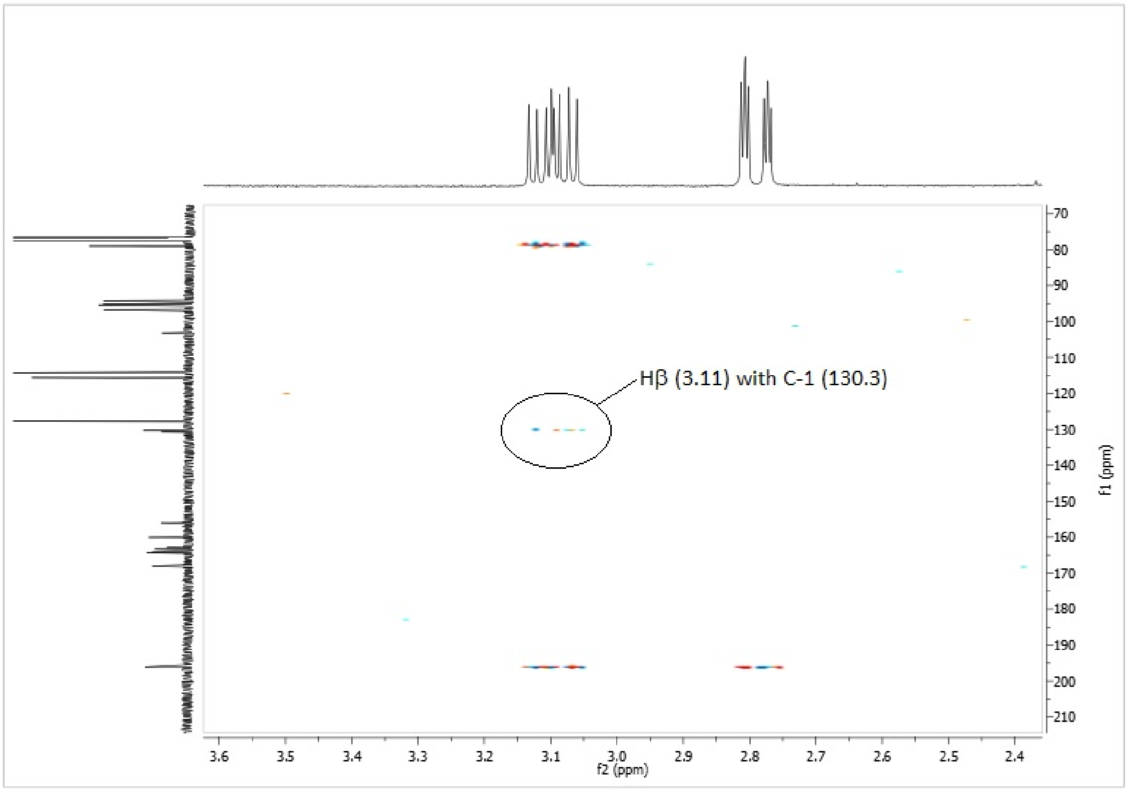

Figure SI-6a: gHMBC spectrum of compound **7A**; Correlation between H-1 (3.11) and C1 (130,3) in compound **7A.**

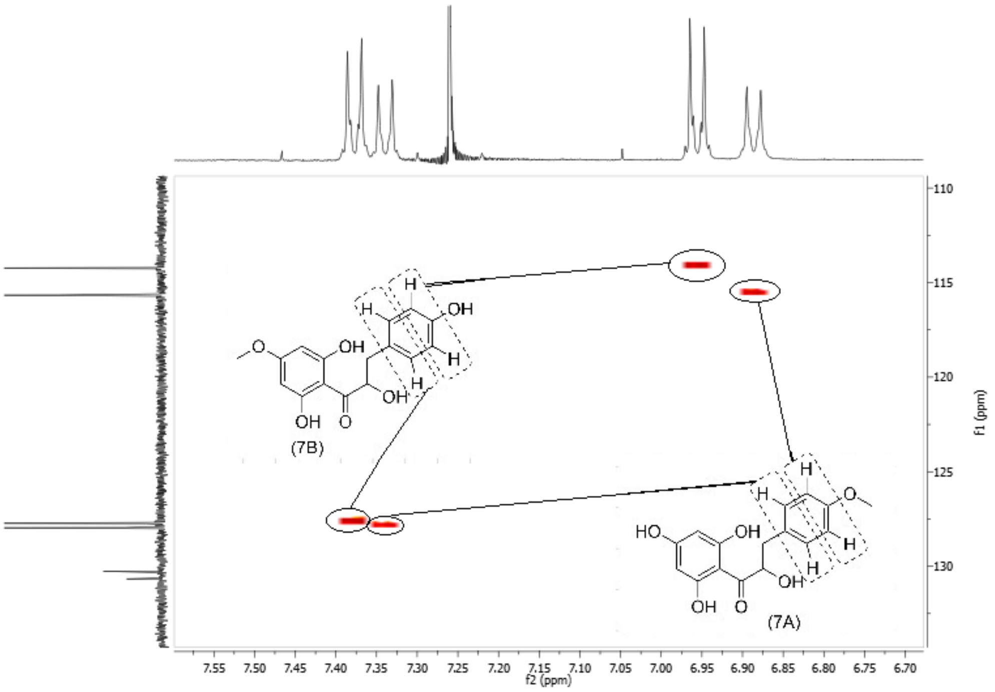

Figure SI-6b: gHMBC spectrum of compound **7A**; Correlation between H2/H6 with C2/C6 and H3/H5 with C3/C5 in compounds**7A** and **7B.**

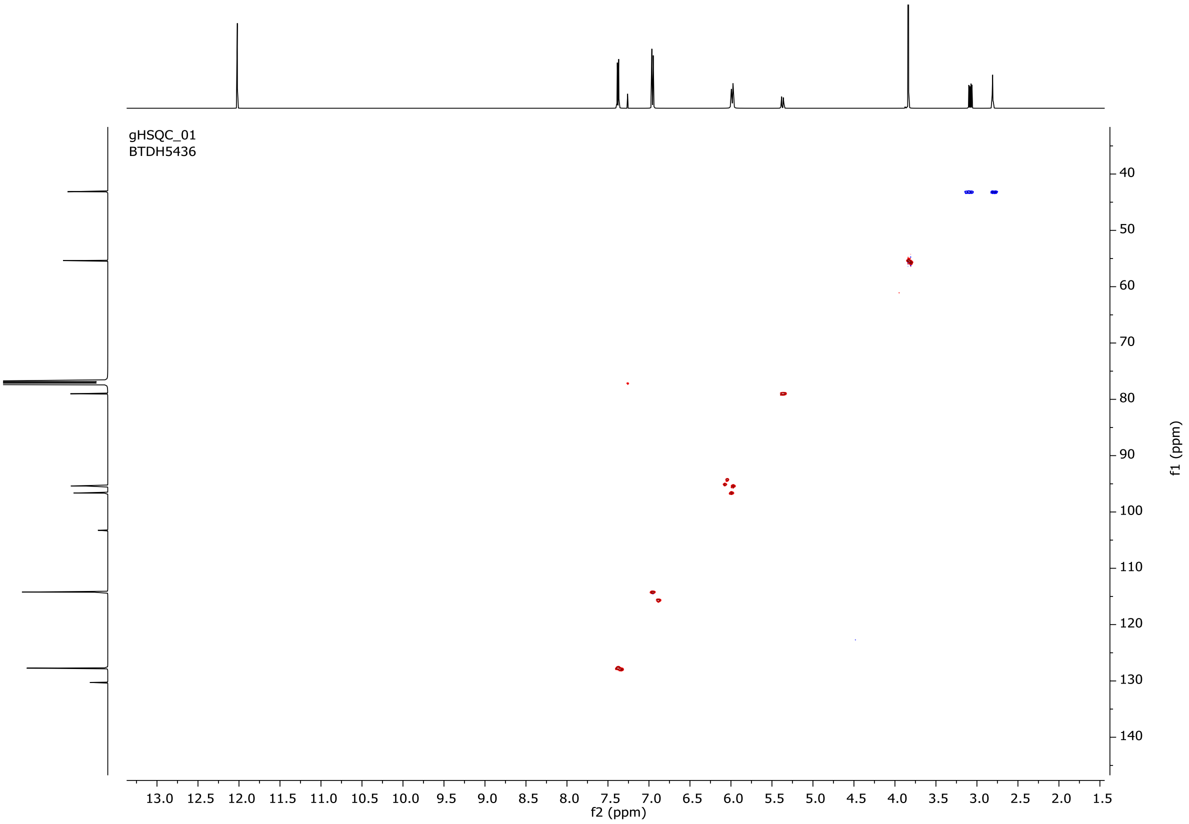

Figure SI- 7: gHSQC spectrum of compound **7A** in $CDCl_3$.

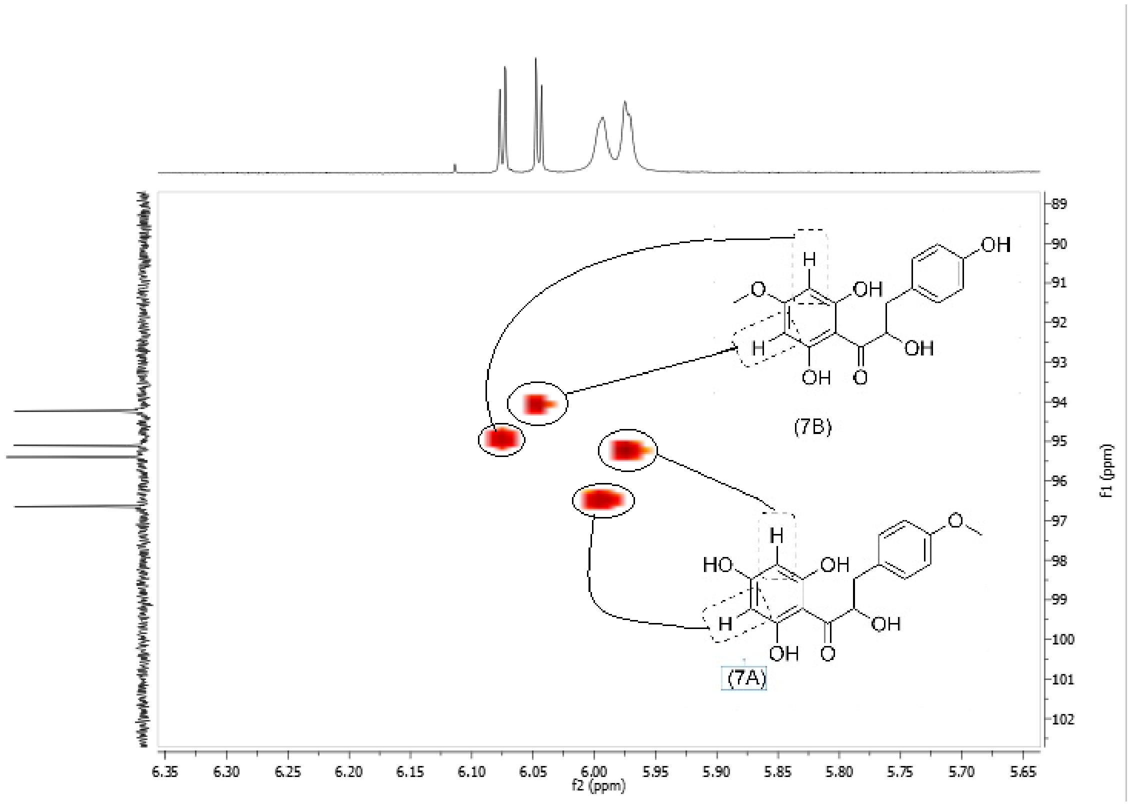

Figure SI- 7a: gHSQC spectrum of compound **7A**;HSQC correlations between H3’(5.99) with C3’ (96.65) and H5’(5.98) with C5’ (95.32) in compound **7A** and correlations between H3’(6.08) with C3’ (95.09) and H5’(6.05) with C5’ (94.19) in compound **7B**.

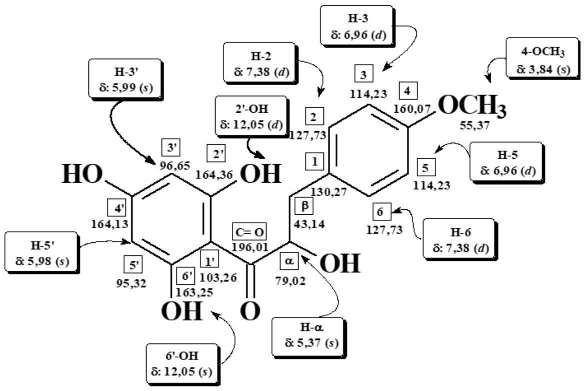

7A

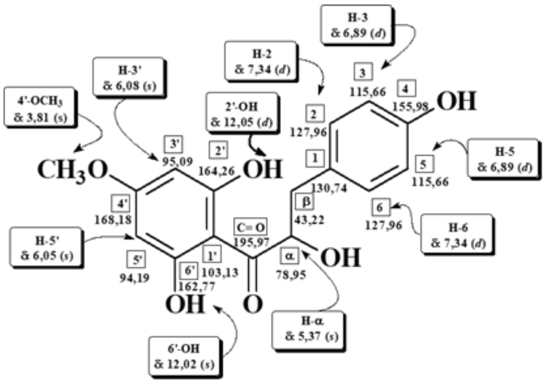

7B

Figure SI- 7b: gHSQC correlations in **7A** and **7B**.

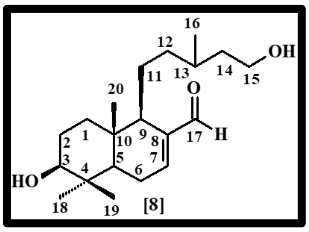

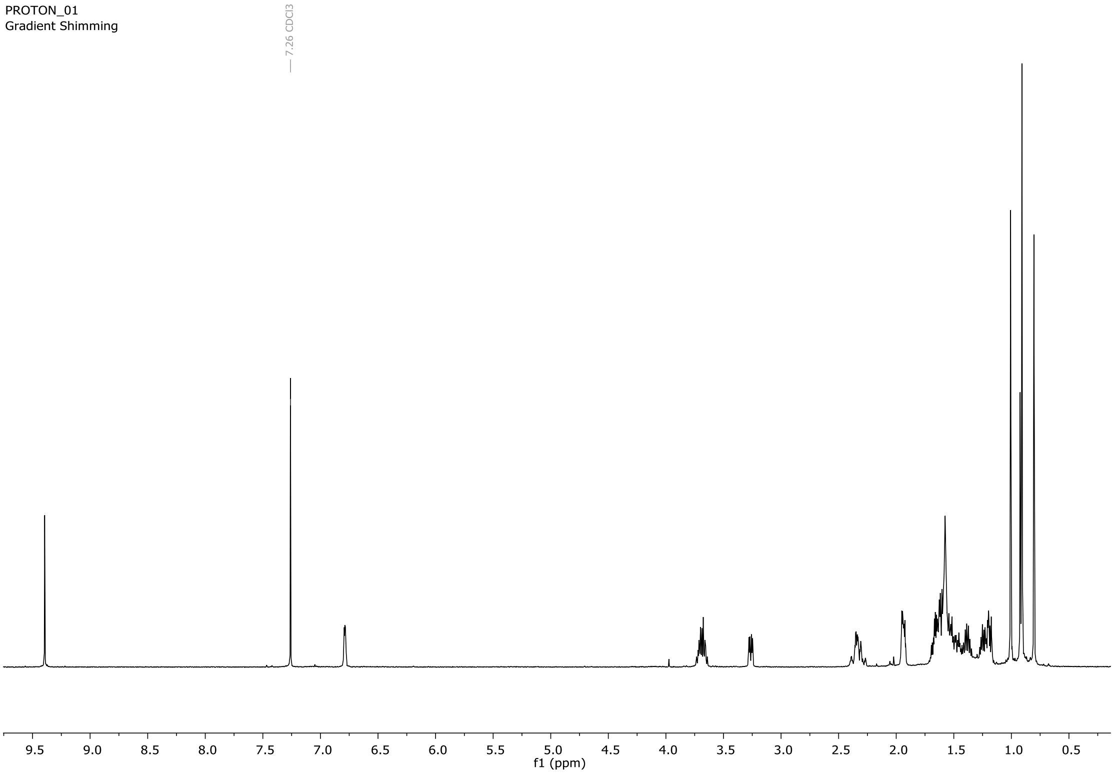

Figure SI- 8: ^{1}H-NMR spectrum of compound **8** in $CDCl_3$ (400MHz).

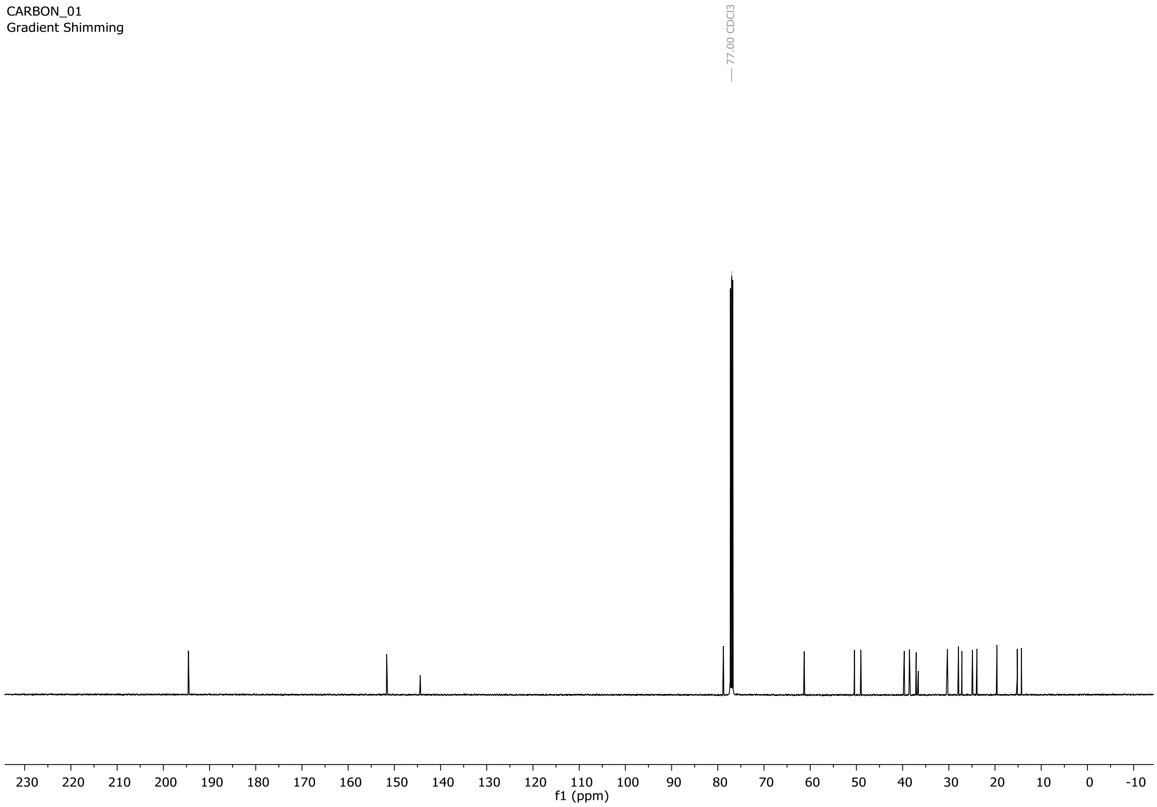

Figure SI- 9: ^{13}C- NMR spectrum of compound **8** in $CDCl_3$ (100MHz).

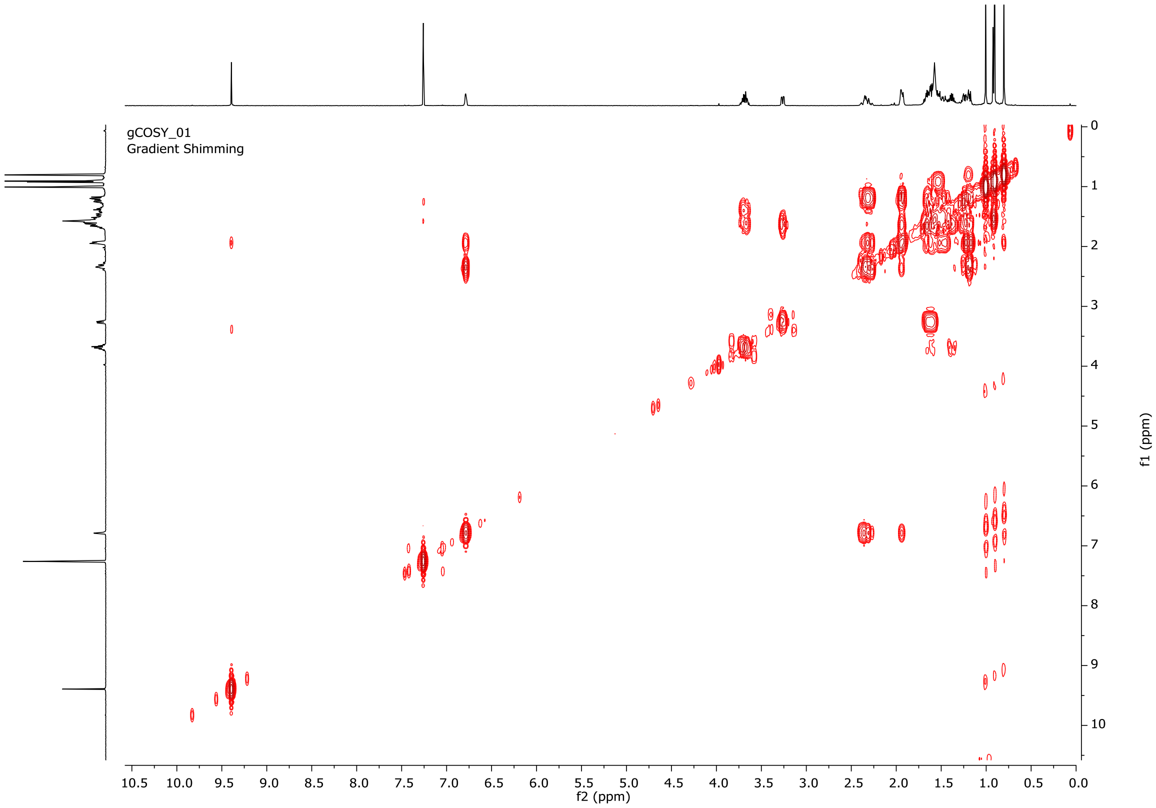

Figure SI-10: gCOSY spectrum of compound **8** in $CDCl_3$ (400MHz).

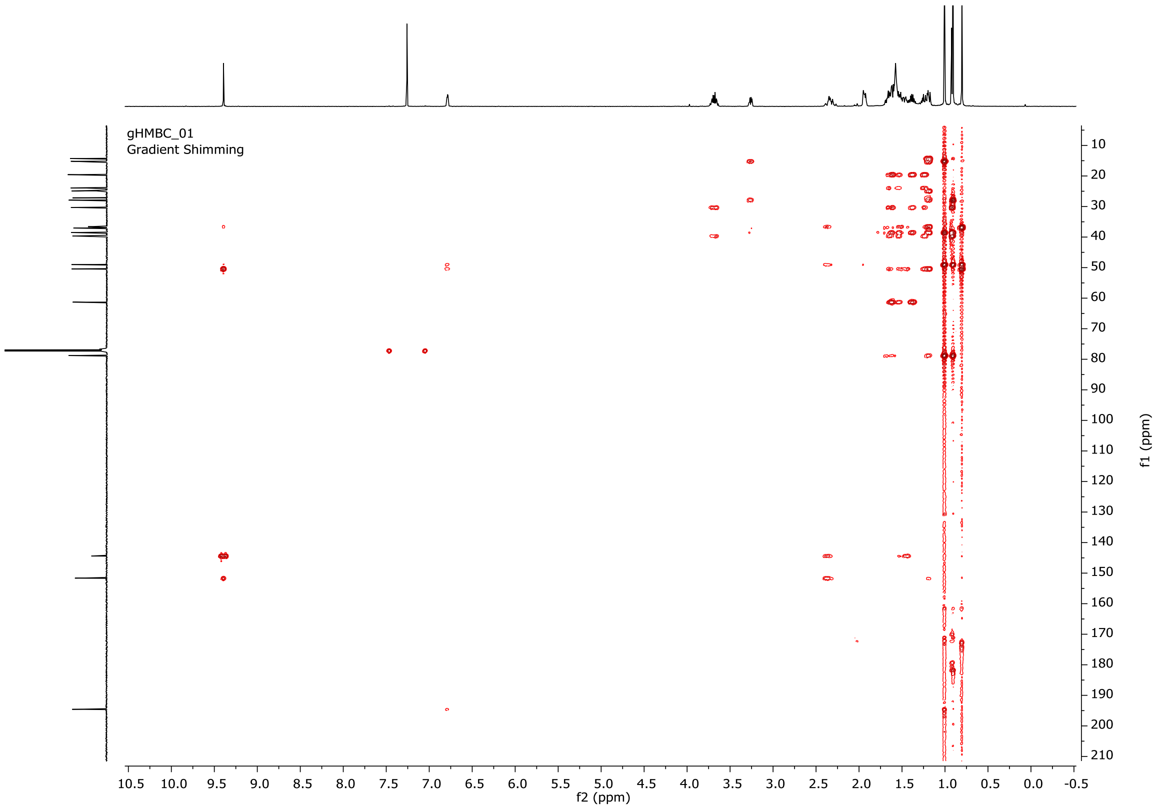

Figure SI-11: gHMBC spectrum of compound **8** in $CDCl_3$.

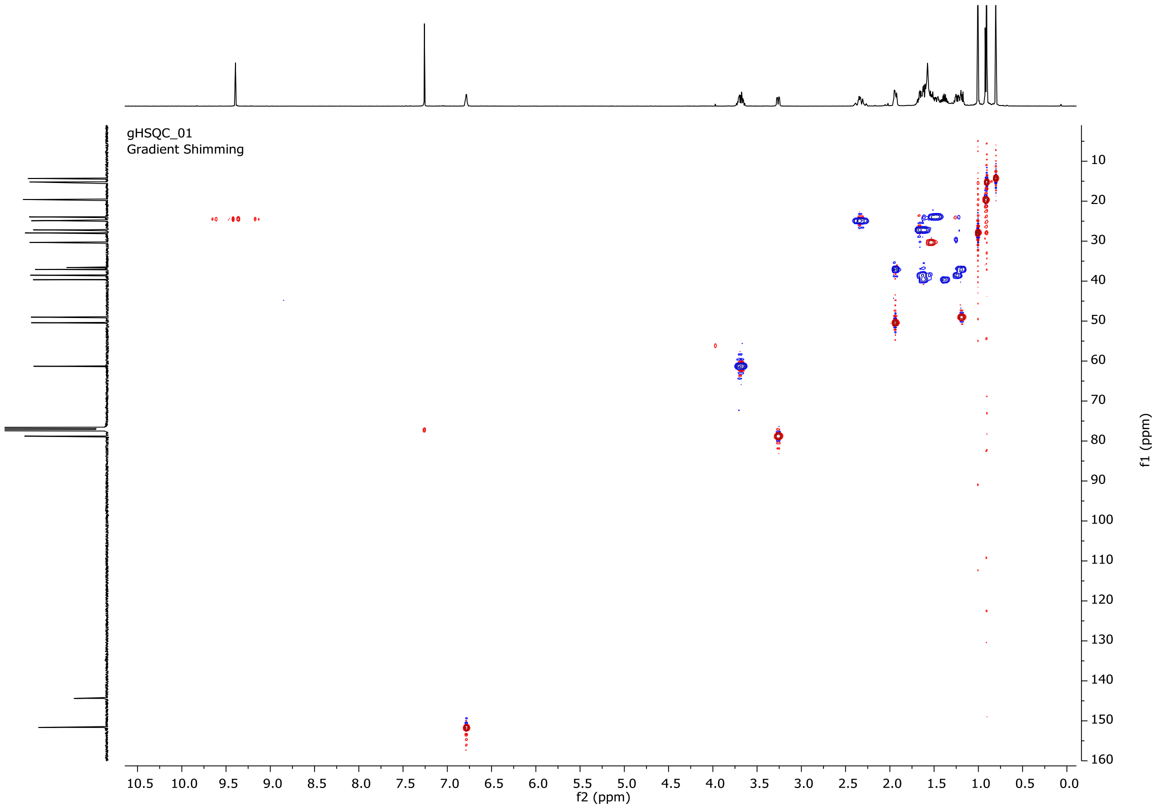

Figure SI-12: gHSQC spectrum of compound **8** in $CDCl_3$.

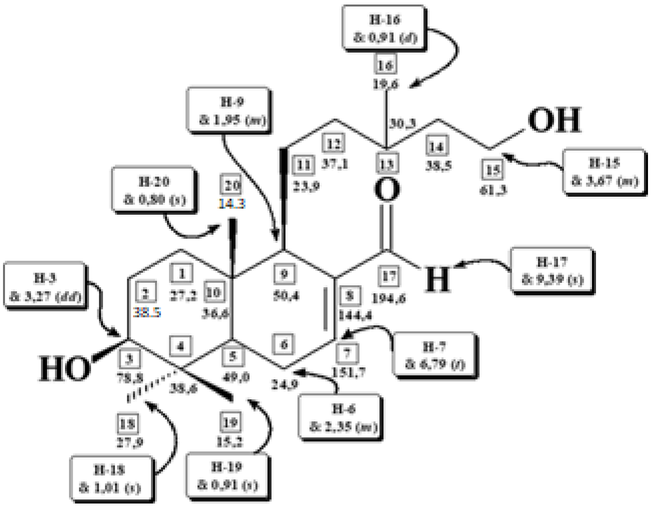

8

Figure SI- 12a: gHSQC correlations of compound **8**.

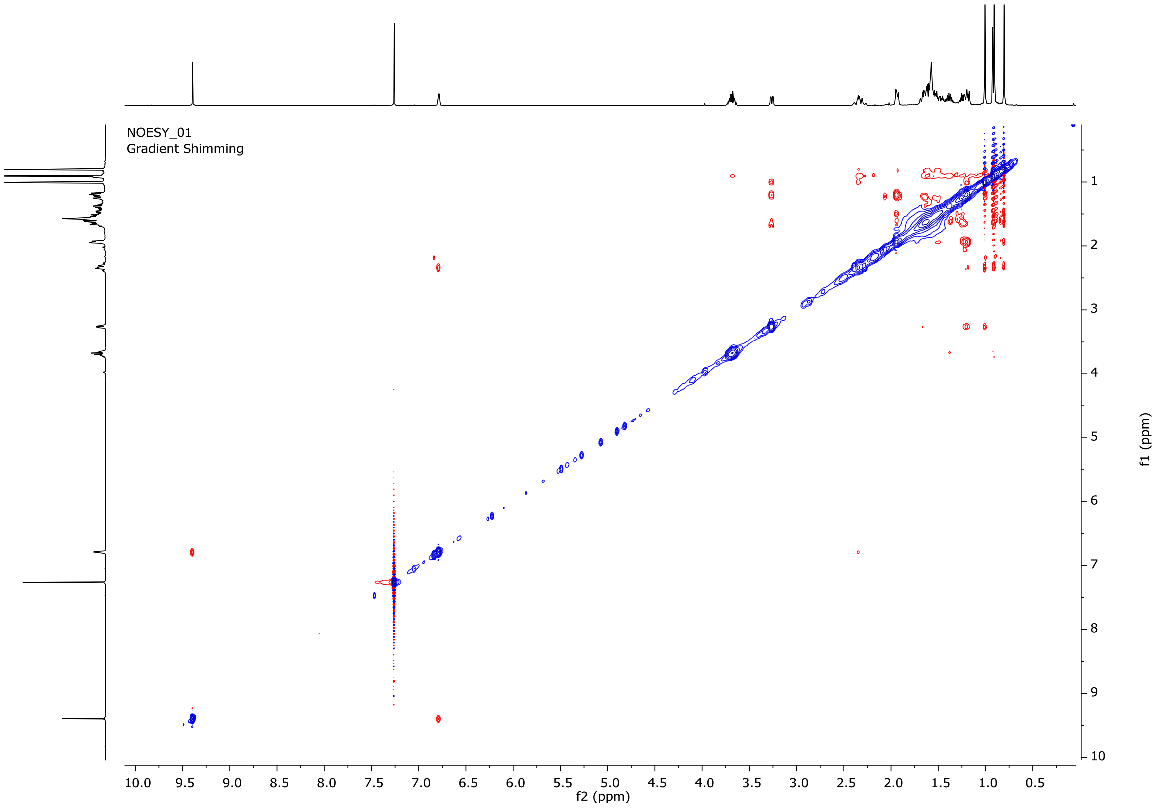

Figure SI-13: NOESY spectrum of compound **8** in $CDCl_3$.

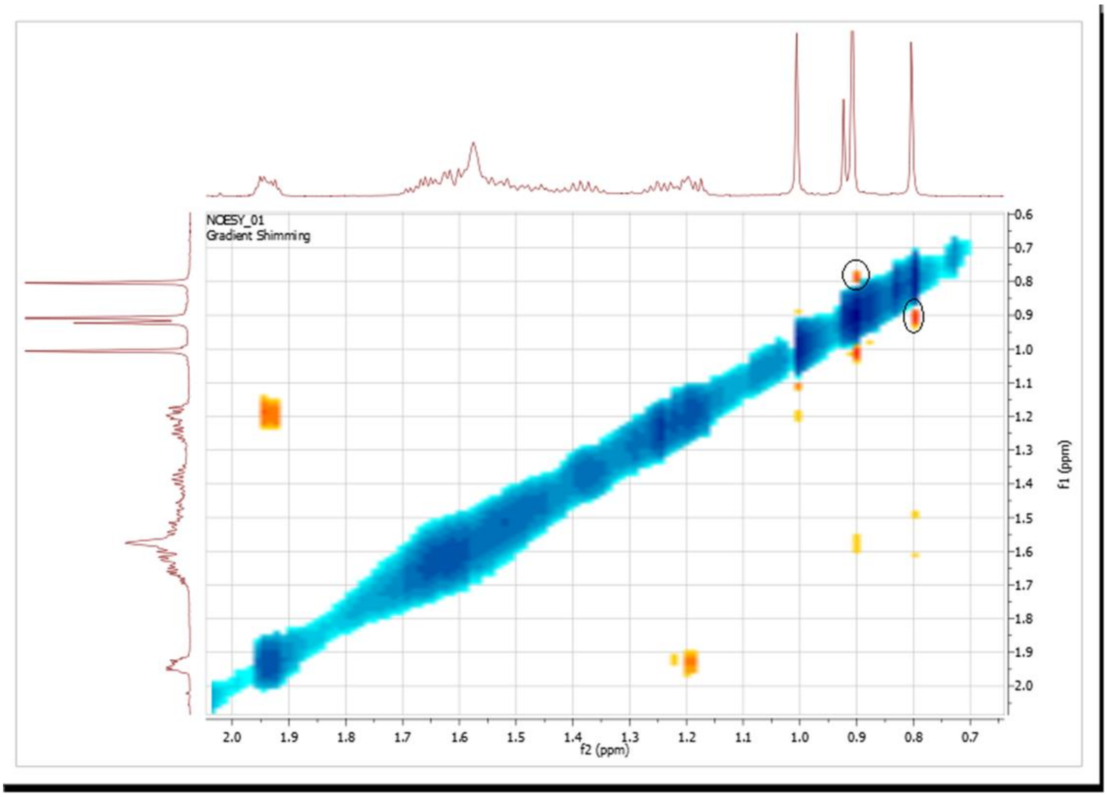

Figure SI-13a: NOESY spectrum of compound **8**; Correlation between H19 and H20.

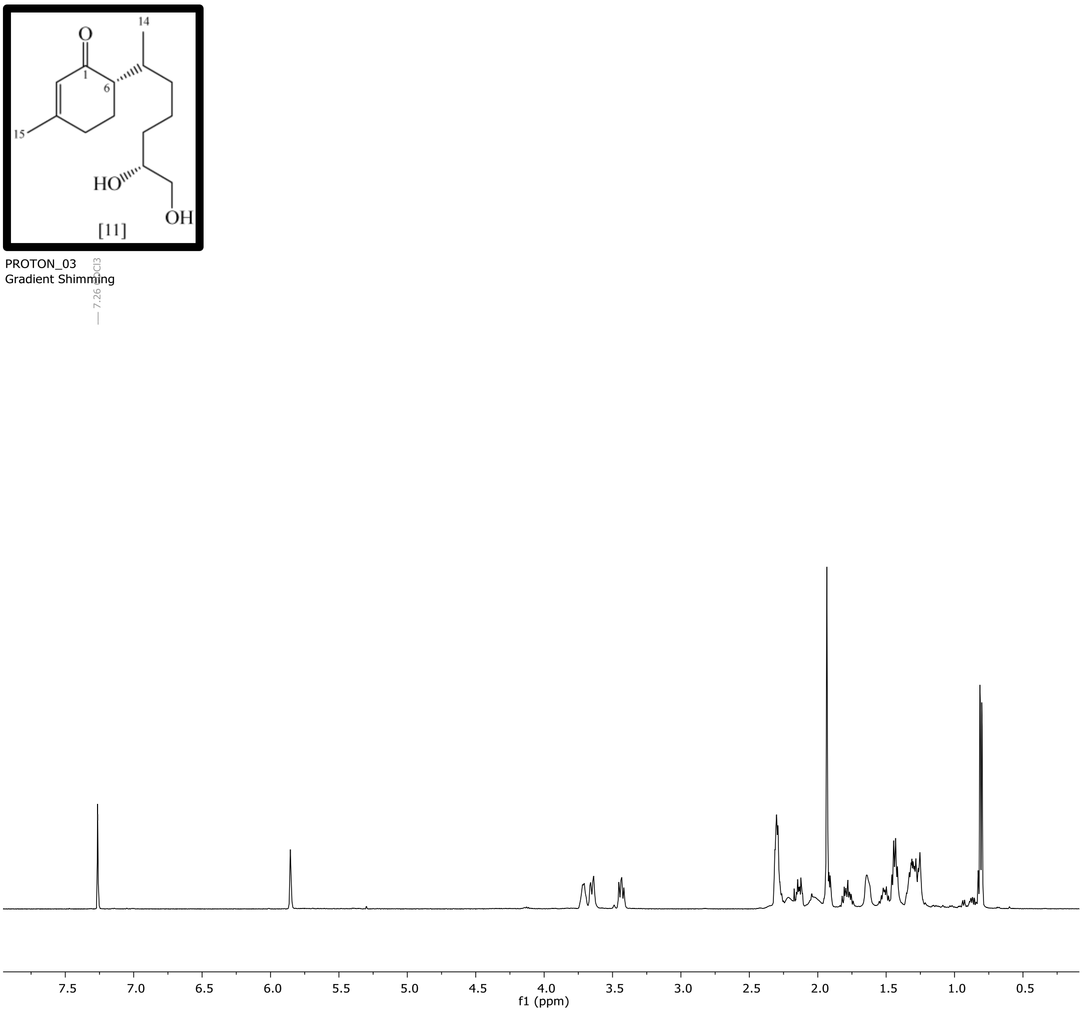

Figure SI-14: ^{1}H-NMR spectrum of compound **11** in $CDCl_3$ (400MHz).

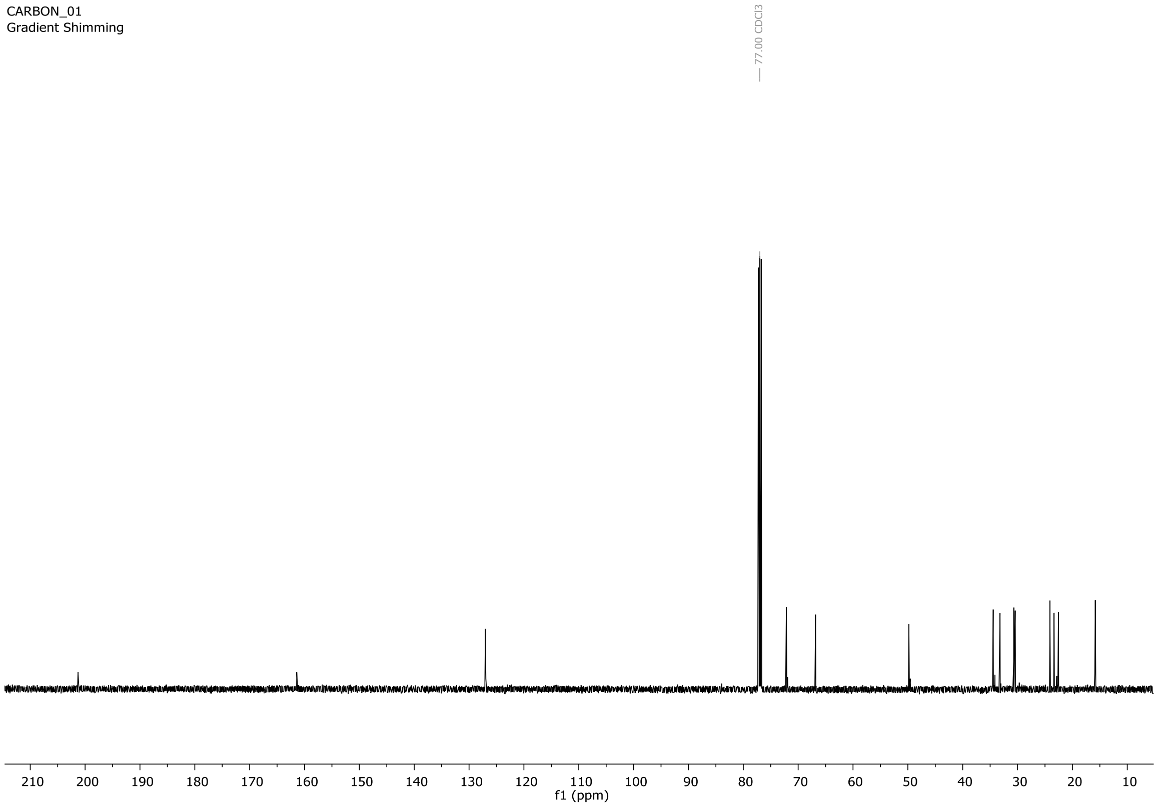

Figure SI-15: ^{13}C- NMR spectrum of compound **11** in $CDCl_3$ (100MHz).

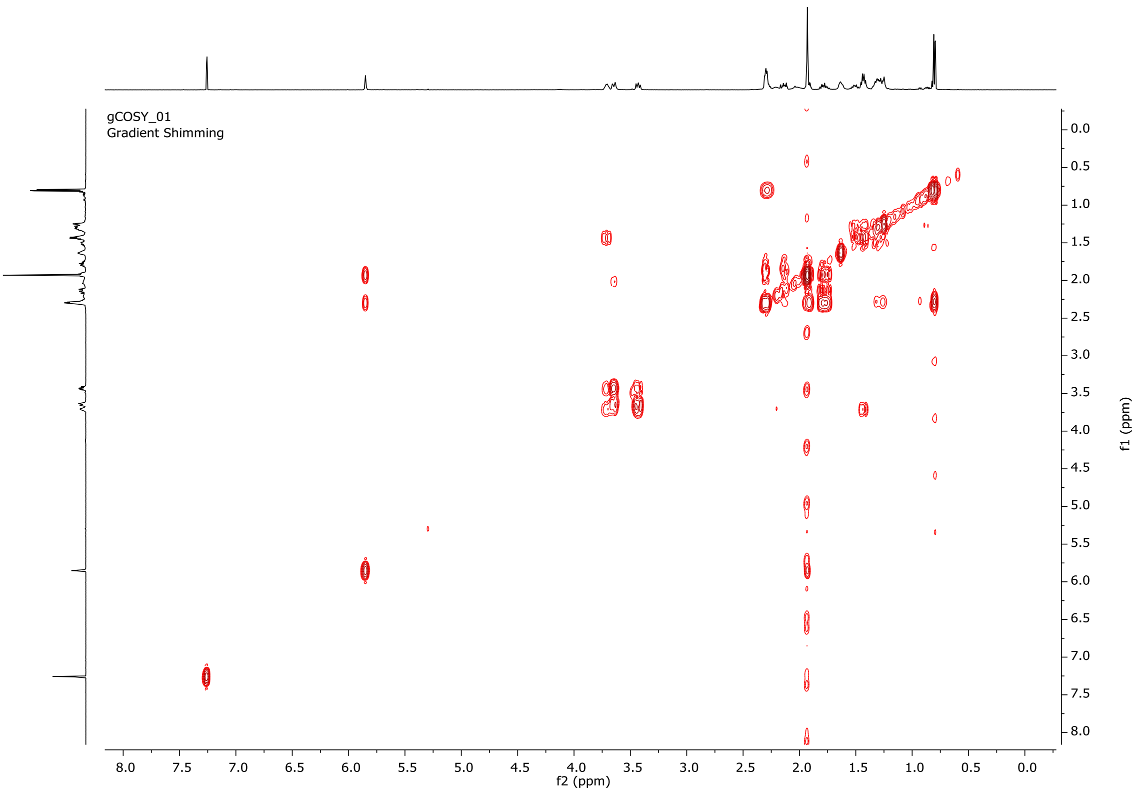

Figure SI-16: gCOSY spectrum of compound **11** in $CDCl_3$.

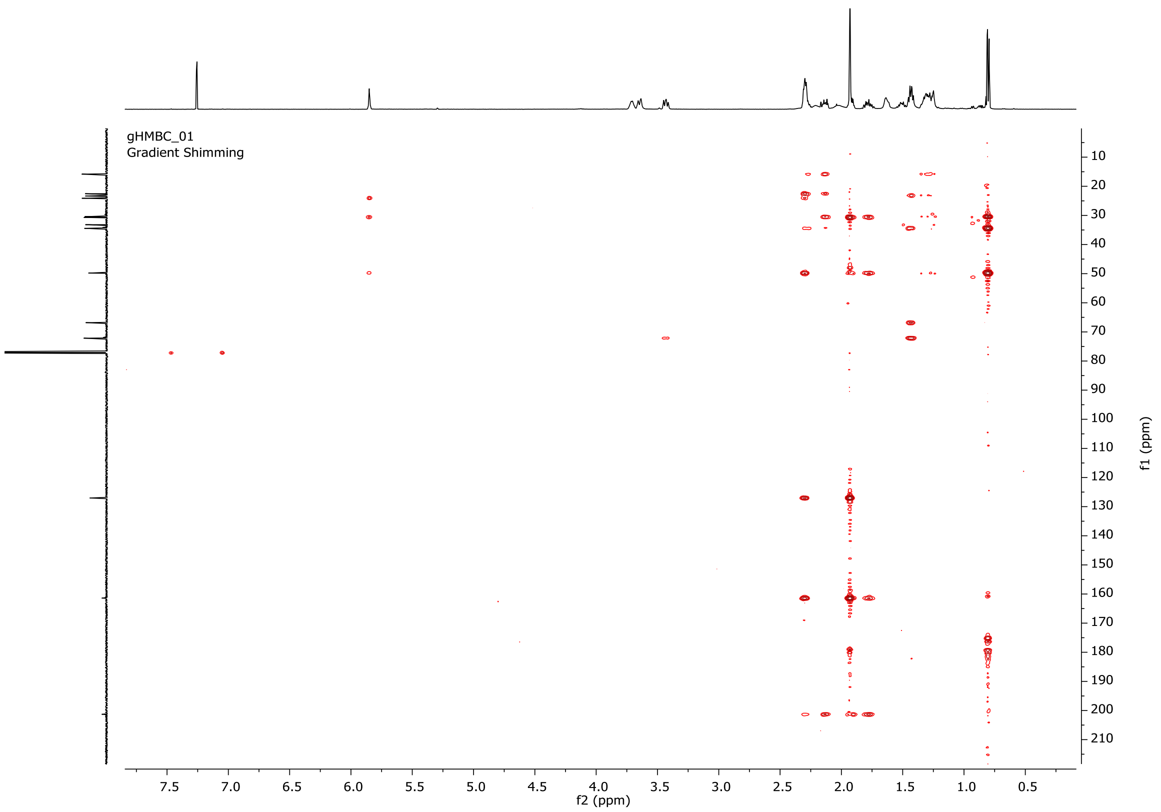

Figure SI-17: gHMBC spectrum of compound **11** in $CDCl_3$.

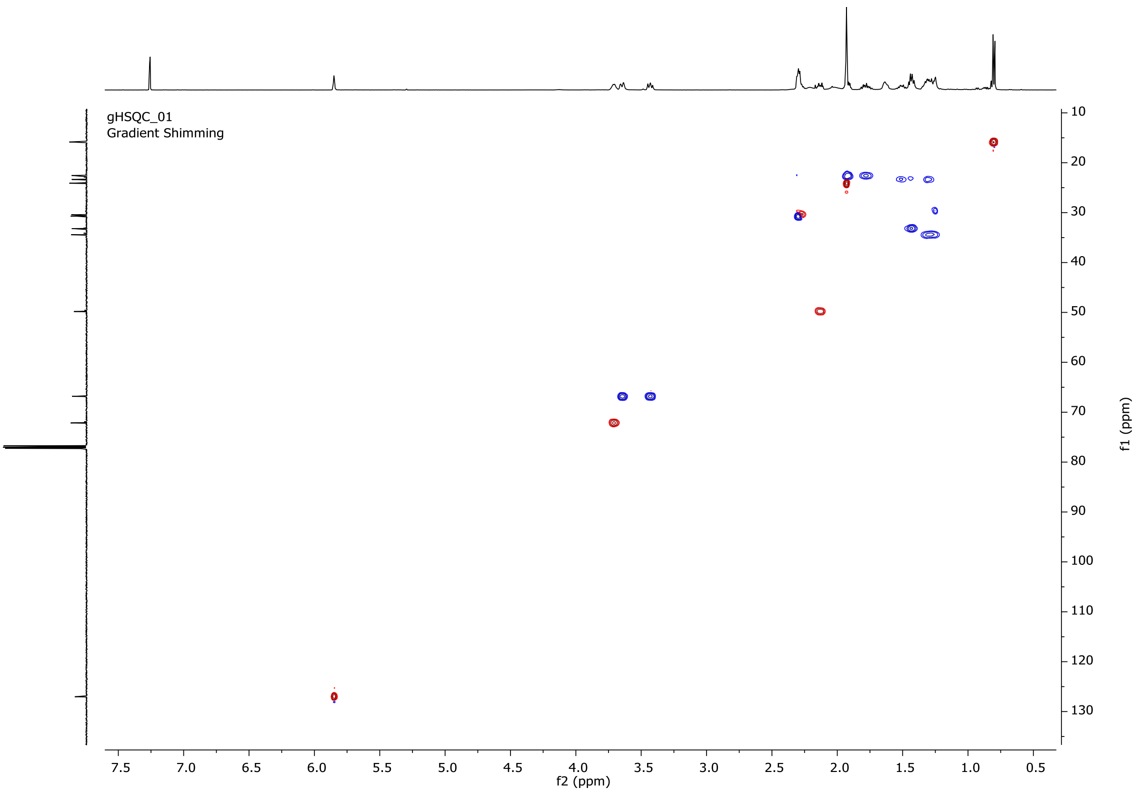

Figure SI-18: gHSQC spectrum of compound **11** in $CDCl_3$.

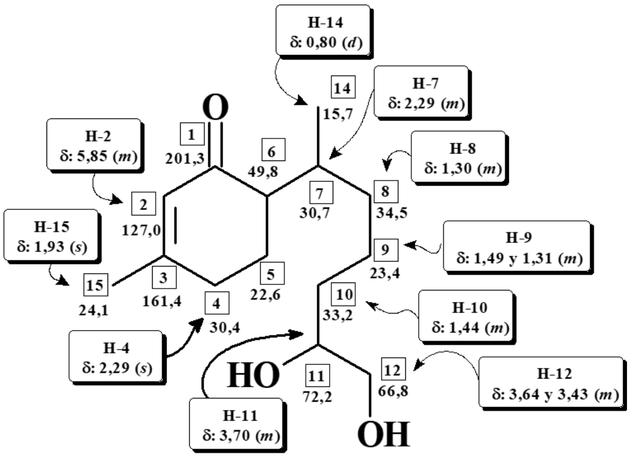

Figure SI-18a: gHSQC correlations of compound **11**.

2- Antifungal activity against Botrytis cinerea UCA 992. Microplate bioassay. Compound 1, 2, 3, 4, 5, 6, 8, 10 and 11

SI- 19- Nevadensin (1).

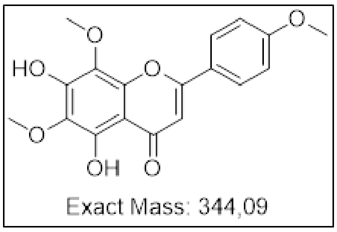

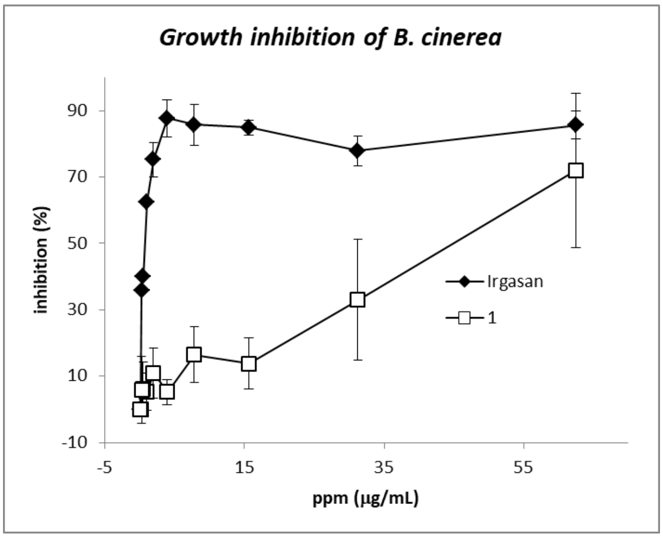

	IC50 (ppm)	57.03
EC50 Lower (ppm)		27.51
EC50 Upper (ppm)		118.2
	IC50 (µM)	165.70
EC50 Lower (µM)		80,0
EC50 Upper (µM)		343,5

Conc. (ppm)	62,50	31,25	15,63	7,81	3,91	1,95	0,98	0,49	0,24	0,12	0,06
Inhibition (%)	72,05	32,97	13,74	16,44	5,17	10,90	5,19	6,12	5,83	0,00	0,00
Standard deviation	23,30	18,21	7,72	8,46	3,68	7,65	5,63	8,06	10,10	0,00	0,00

Log EC_{50}= 1.756

EC_{50} = 57.03 ppm

EC_{50} lower 95% confidence limit = 27.51 ppm

EC_{50} upper 95% confidence limit = 118.2 ppm

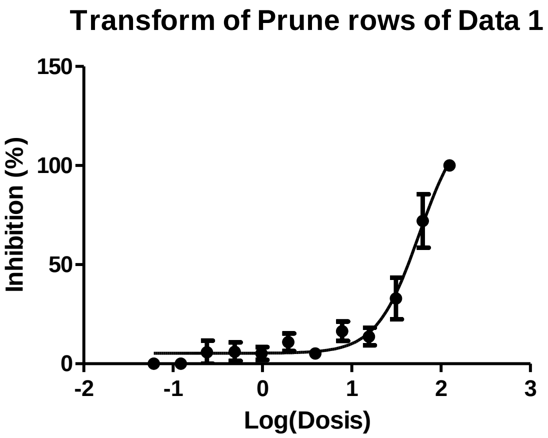

Error estandard LogEC50=0.1551
(debe ser <0.5; EC50 valido)
R^2=0.8659

SI- 20- 4’,7-Dimethoxyapigenin (2).

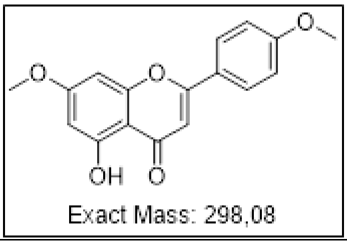

IC50 (ppm)	31.25
EC50 Lower (ppm)	
EC50 Upper (ppm)	
IC50 (µM)	104
EC50 Lower (µM)	
EC50 Upper (µM)	

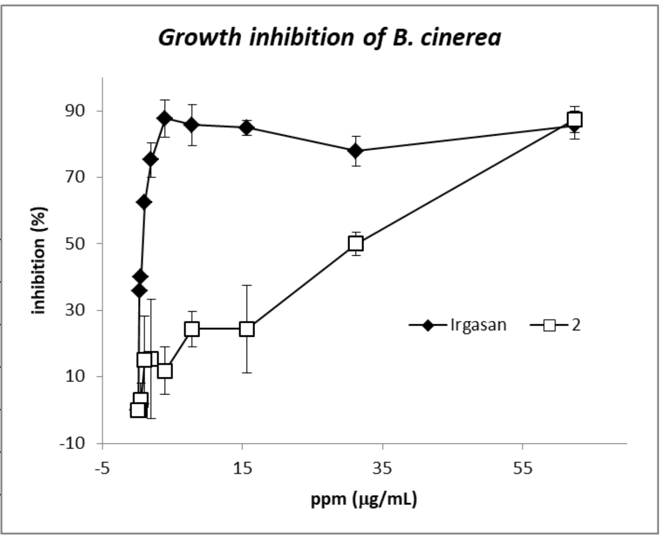

Conc. (ppm)	*62,50*	*31,25*	*15,63*	*7,81*	*3,91*	*1,95*	*0,98*	*0,49*	*0,24*	*0,12*	*0,06*
**Inhibition (%)**	87,42	49,98	24,35	24,34	11,85	15,38	15,10	2,94	0,00	0,00	0,00
**Standard deviation**	3,89	3,62	13,24	5,24	7,17	17,99	13,27	5,09	0,00	0,00	0,00

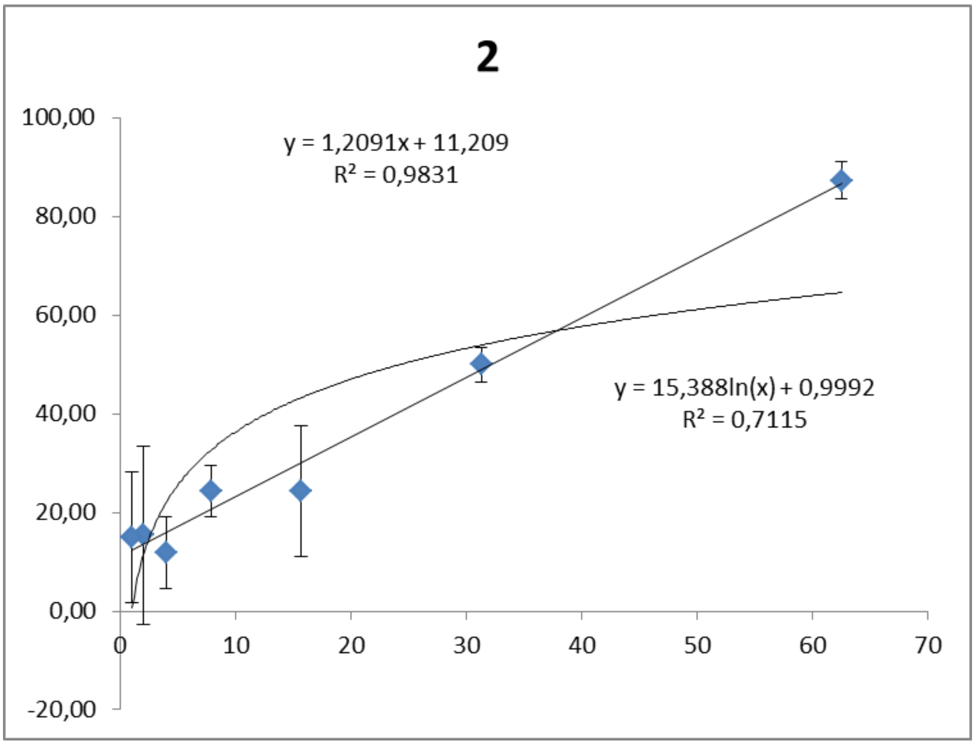

SI-21- Genkwanin (3).

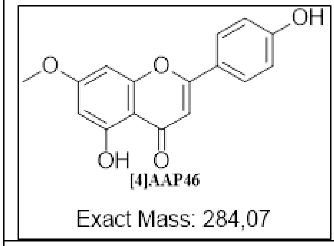

IC50 (ppm)	35.9
EC50 Lower (ppm)	31.61
EC50 Upper (ppm)	40.81
IC50 (µM)	126.4
EC50 Lower (µM)	111.3
EC50 Upper (µM)	143.7

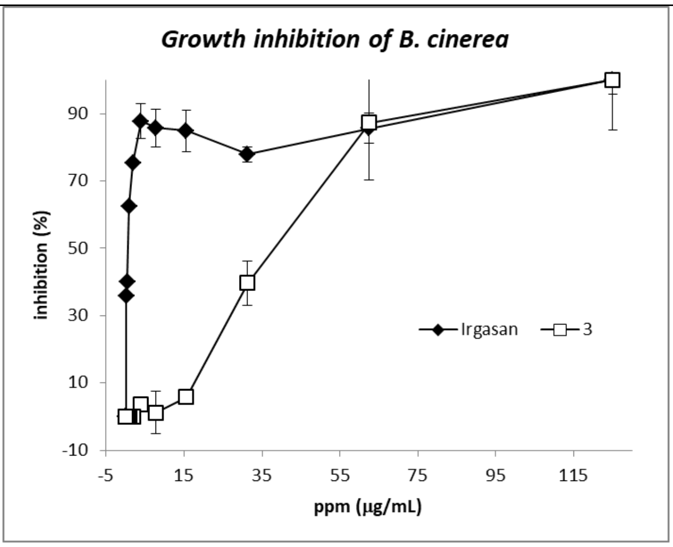

Conc. (ppm)	*62,50*	*31,25*	*15,63*	*7,81*	*3,91*	*1,95*	*0,98*	*0,49*	*0,24*	*0,12*	*0,06*
**Inhibition (%)**	87,28	39,64	5,77	1,16	3,69	0,00	0,00	0,00	0,00	0,00	0,00
**Standard deviation**	14,74	17,09	6,53	2,02	6,39	0,00	0,00	0,00	0,00	0,00	0,00

Log EC_{50}= 1.555

EC_{50} = 35.92 ppm

EC_{50} lower 95% confidence limit = 31.61 ppm

EC_{50} upper 95% confidence limit = 40.81 ppm

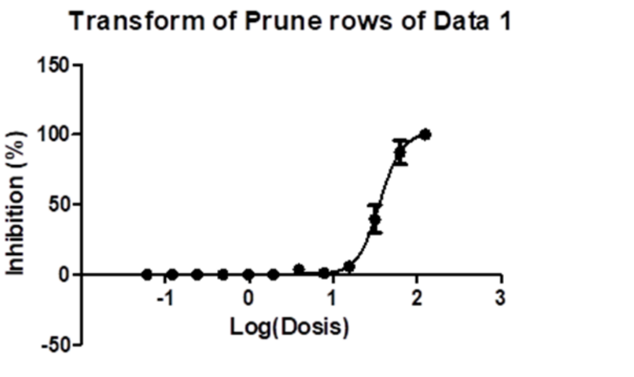

Error estandard LogEC50=0.02719
(debe ser <0.5; EC50 valido)
R^2=0.9678

SI- 22- Cirsimaritin (4).

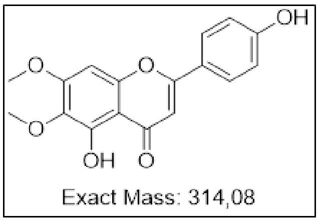

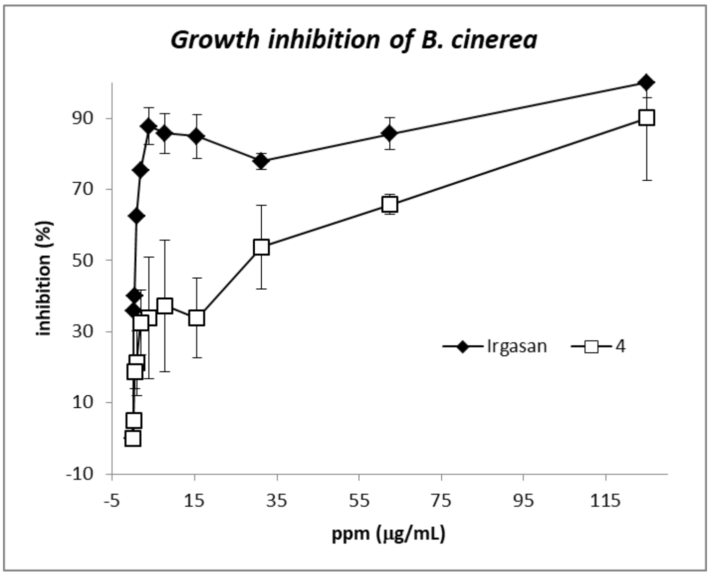

	IC50 (ppm)	*38.9*
EC50 Lower (ppm)		
EC50 Upper (ppm)		
	**IC50 (µM)**	123.9
EC50 Lower (µM)		
EC50 Upper (µM)		

Conc. (ppm)	*62,50*	*31,25*	*15,63*	*7,81*	*3,91*	*1,95*	*0,98*	*0,49*	*0,24*	*0,12*	*0,06*
**Inhibition (%)**	65,68	53,76	33,84	37,32	33,86	32,60	21,21	18,73	4,95	0,00	0,00
**Standard deviation**	17,50	2,80	11,70	11,14	18,47	17,02	9,13	9,09	4,73	0,00	0,00

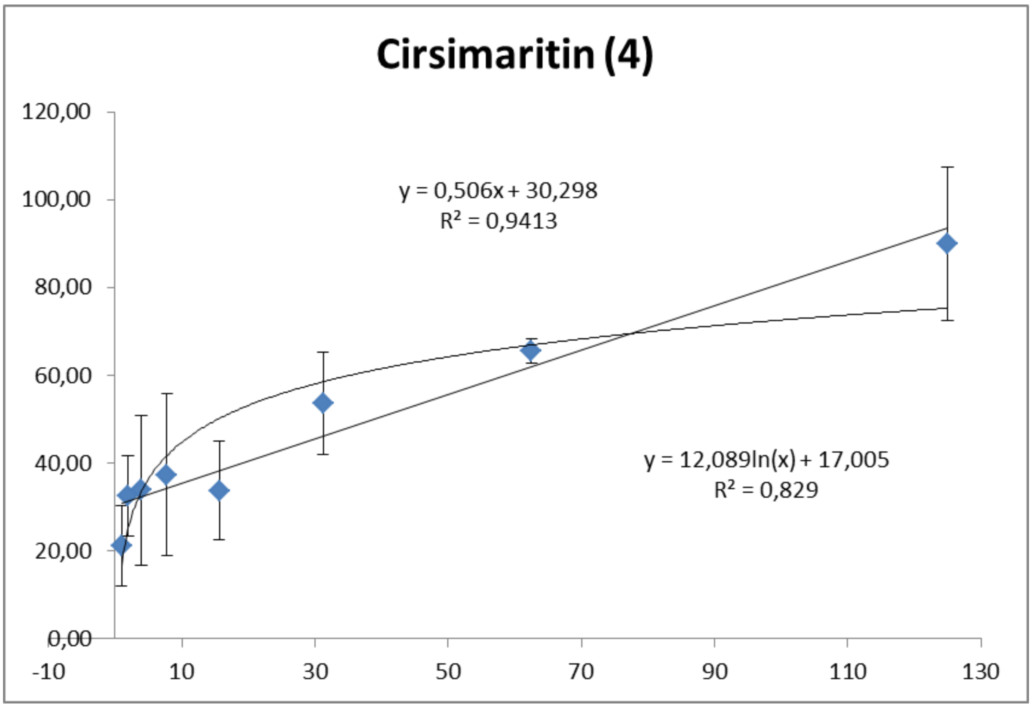

SI- 23- Salvigenin (5).

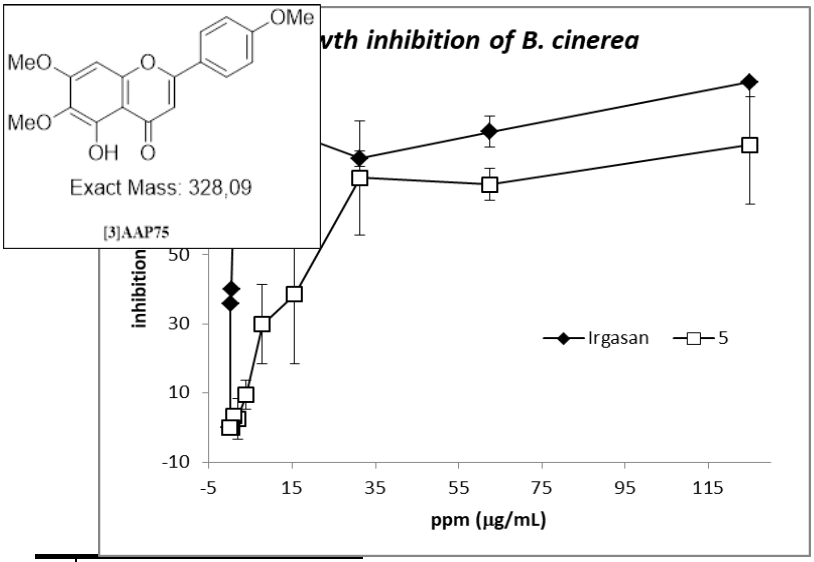

IC50 (ppm)	*13.49*
EC50 Lower (ppm)	8.69
EC50 Upper (ppm)	20.94
**IC50 (µM)**	41.1
EC50 Lower (µM)	26.5
EC50 Upper (µM)	63.8

Conc. (ppm)	62,50	31,25	15,63	7,81	3,91	1,95	0,98	0,49	0,24	0,12	0,06
**Inhibition (%)**	70,31	72,27	38,61	29,94	9,56	2,42	3,36	0,00	0,00	0,00	0,00
**Standard deviation**	17,35	4,60	16,65	20,08	11,41	4,19	5,81	0,00	0,00	0,00	0,00

Log EC_{50}= 1.130

EC_{50} = 13.49 ppm

EC_{50} lower 95% confidence limit = 8.688 ppm

EC_{50} upper 95% confidence limit = 20.94 ppm

Transform of Prune rows of Data 1

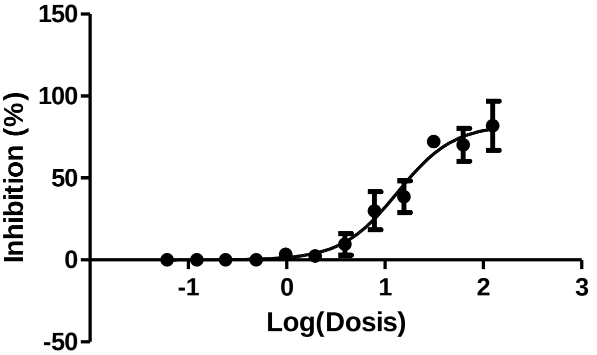

Error estandard LogEC50=0.0936
(debe ser <0.5; EC50 valido)
R^2=0.9017

SI- 24- Galangustin (6).

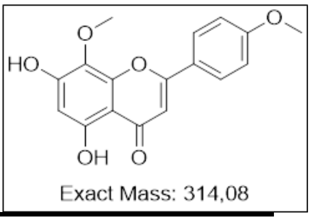

	IC50 (ppm)	*14.06*
EC50 Lower (ppm)		9.41
EC50 Upper (ppm)		21.01
	**IC50 (μM)**	44.8
EC50 Lower (μM)		30.0
EC50 Upper (μM)		66.9

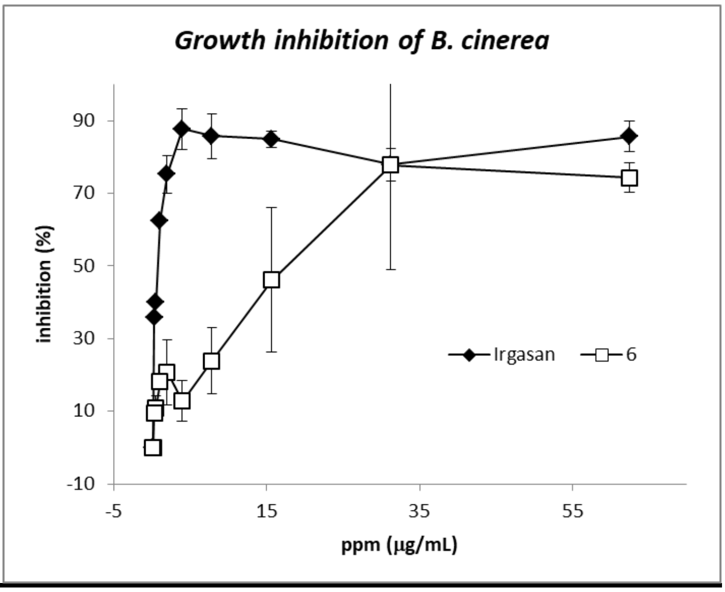

Conc. (ppm)	62,50	31,25	15,63	7,81	3,91	1,95	0,98	0,49	0,24	0,12	0,06

**Inhibition (%)**	74,32	77,75	46,17	23,89	12,78	20,79	18,14	10,97	9,54	0,00	0,00
**Standard deviation**	16,89	4,08	28,83	19,90	9,10	5,67	8,96	1,47	3,23	0,00	0,00

Log EC_{50}= 1.148

EC_{50} = 14.06 ppm

EC_{50} lower 95% confidence limit = 9.408 ppm

EC_{50} upper 95% confidence limit = 21.01 ppm

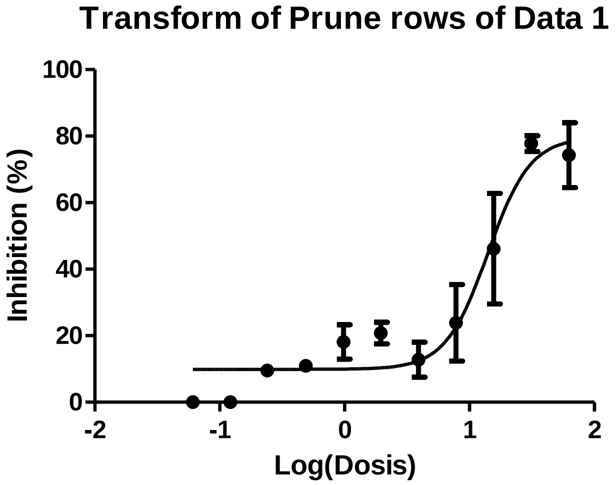

Error estandard LogEC50=0.08533
(debe ser <0.5; EC50 valido)
R^2=0.8164

__SI- 25- 3β,15-Dihydroxylabdan-7-en-17-al (8).__

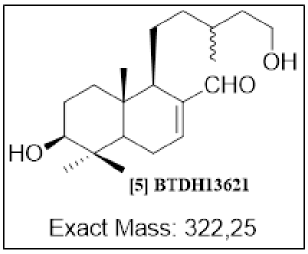

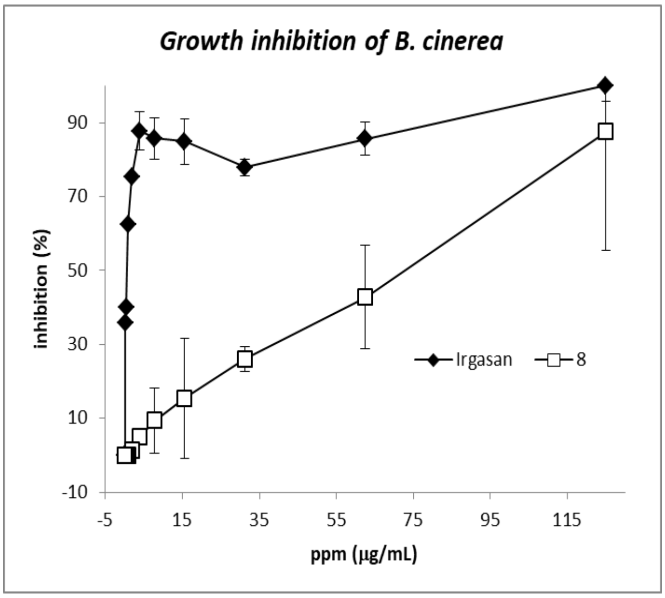

	IC50 (ppm)	*70.04*

EC50 Lower (ppm)	
EC50 Upper (ppm)	
**IC50 (μM)**	246.6
EC50 Lower (μM)	
EC50 Upper (μM)	

Conc. (ppm)	125,00	62,50	31,25	15,63	7,81	3,91	1,95	0,98	0,49	0,24	0,12
**Inhibition (%)**	87,54	42,80	26,03	15,38	9,37	5,07	1,30	0,00	0,00	0,00	0,00
**Standard deviation**	5,50	32,02	14,00	3,41	16,23	8,78	2,25	0,00	0,00	0,00	0,00

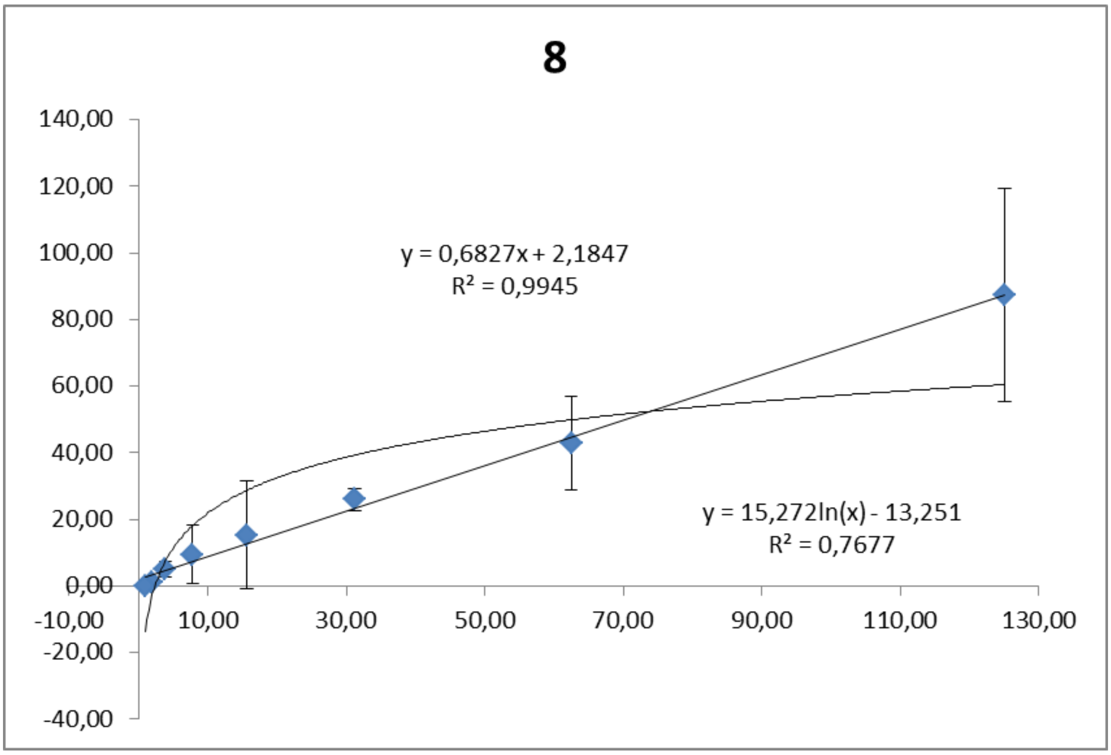

SI- 26- 1,2-Dyhidrosenedigital-2-ona (10).

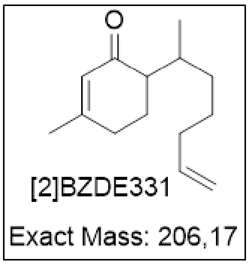

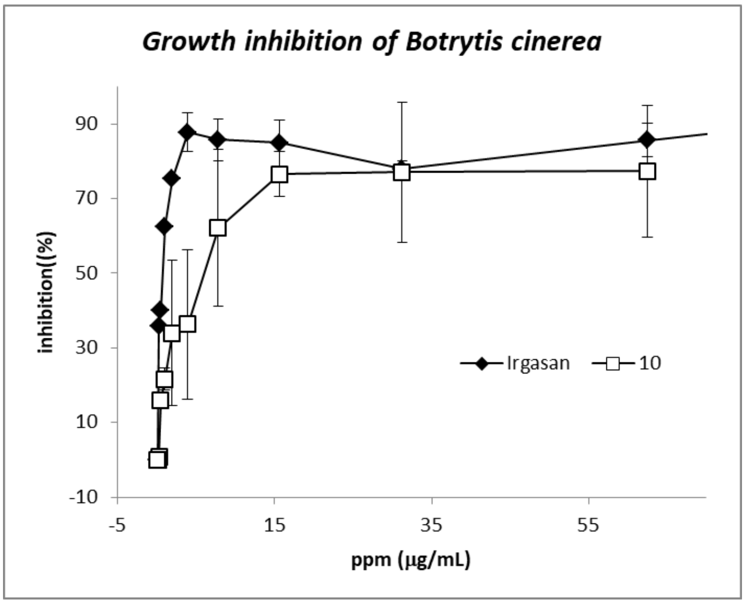

	IC50 (ppm)	*3.12*
EC50 Lower (ppm)		1.36
EC50 Upper (ppm)		7.15
	**IC50 (µM)**	15.2
EC50 Lower (µM)		6.6
EC50 Upper (µM)		34.7

Conc. (ppm)	62,50	31,25	15,63	7,81	3,91	1,95	0,98	0,49	0,24	0,12	0,06
**Inhibition (%)**	77,31	77,05	76,58	62,14	36,31	33,94	21,62	15,94	0,66	0,00	0,00
**Standard deviation**	20,87	17,58	18,83	5,93	21,12	20,07	19,54	2,86	1,14	0,00	0,00

Log EC_{50}= 0.4949

EC_{50} = 3.126 ppm

EC_{50} lower 95% confidence limit = 1.365 ppm

EC_{50} upper 95% confidence limit = 7.156 ppm

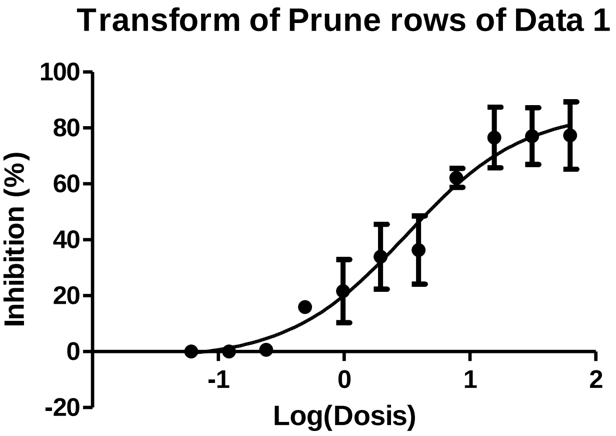

Error estandard LogEC50=0.1759
(debe ser <0.5; EC50 valido)
R^2=0.8468

SI- 27- 13-nor-11,12-Dihydroxybisabol-2-enone (11).

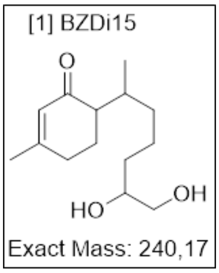

	IC50 (ppm)	*59.07*
EC50 Lower (ppm)		53.79
EC50 Upper (ppm)		64.87
	**IC50 (μM)**	246.0
EC50 Lower (μM)		224.0
EC50 Upper (μM)		270.1

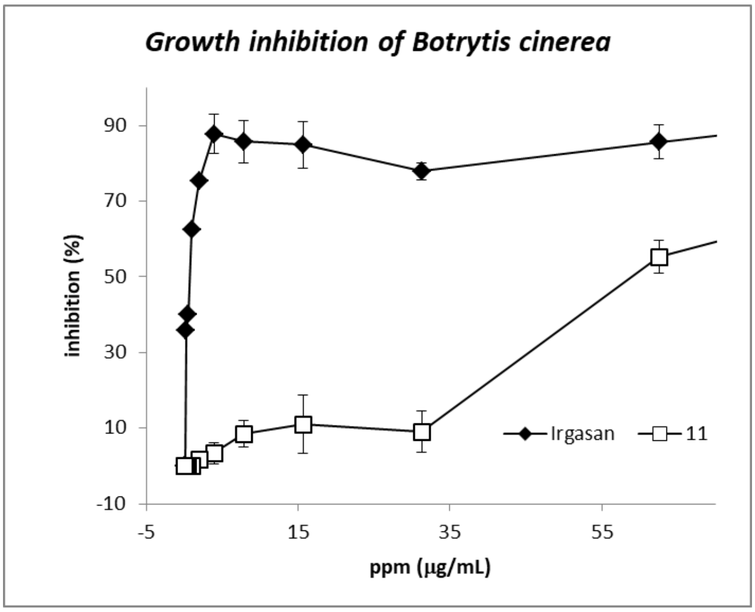

Conc. (ppm)	125,00	62,50	31,25	15,63	7,81	3,91	1,95	0,98	0,49	0,24	0,12
**Inhibition (%)**	88,51	55,26	9,04	10,94	8,45	3,34	1,62	0,00	0,00	0,00	0,00
**Standard deviation**	5,04	11,33	4,32	5,57	7,76	3,57	2,81	0,00	0,00	0,00	0,00

Log EC_{50}= 1.771

EC_{50} = 59.07 ppm

EC_{50} lower 95% confidence limit = 53.79 ppm

EC_{50} upper 95% confidence limit = 64.87 ppm

Transform of Prune rows of Data 1

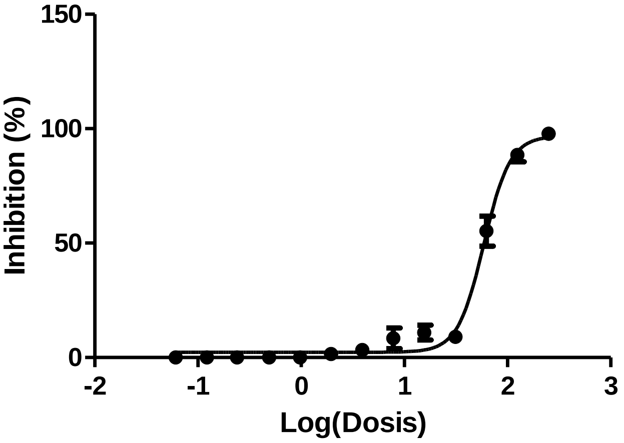

Error estandard LogEC50=0.02001
(debe ser <0.5; EC50 valido)
R^2=0.9748

<u>SI- 28- Plate bioassay.</u> Compound 5

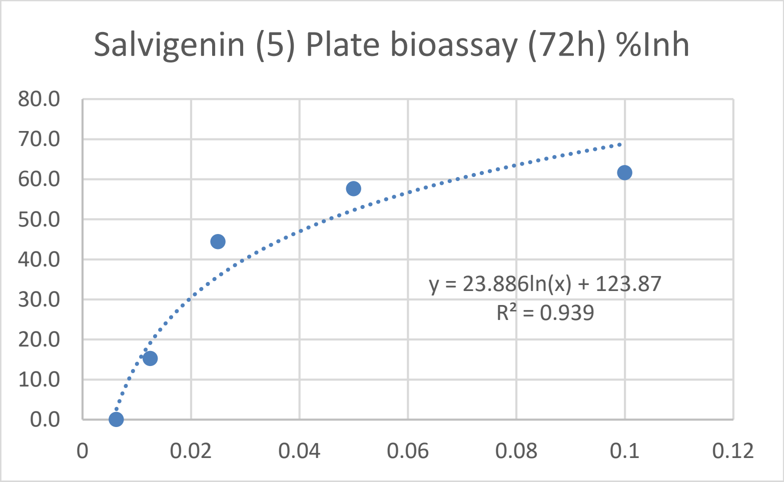

Salvigenin (5) (72h)	
**Conc**	**%Inh**
0,1	61,6
0,05	57,6
0,025	44,4
0,0125	15,2
0,00625	0,0
IC50 (ppm)	*14.89*
**IC50 (μM)**	45.4

SI- 29- Plate bioassay. Compound 10

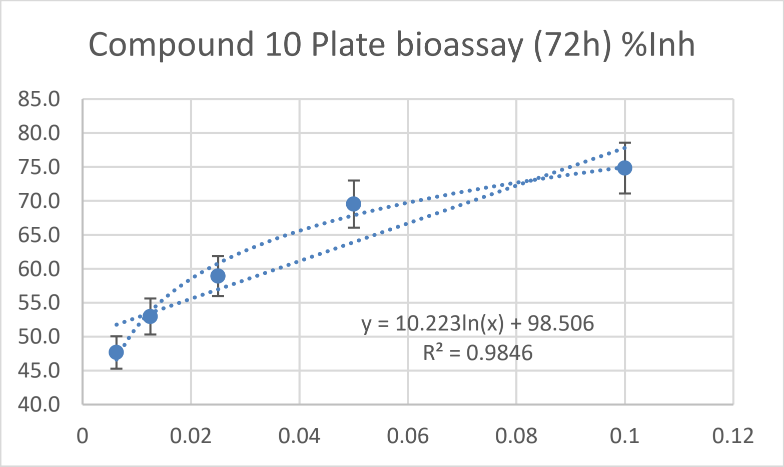

Compound 10 (72h)	
**Conc**	**%Inh**
0,1	74,8
0,05	69,5
0,025	58,9
0,0125	53,0
0,00625	47,7
IC50 (ppm)	*1.79*
**IC50 (μM)**	8.7

SI- 30- Positive control. Microplate Bioassay (irgasan).

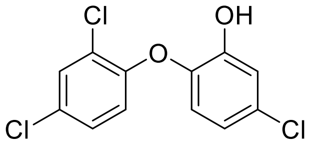

Exact Mass: 287,9512

IC50 (ppm)	***0.23***
EC50 Lower (ppm)	0.19
EC50 Upper (ppm)	0.29
**IC50 (μM)**	0.80
EC50 Lower (μM)	0.65
EC50 Upper (μM)	1.00

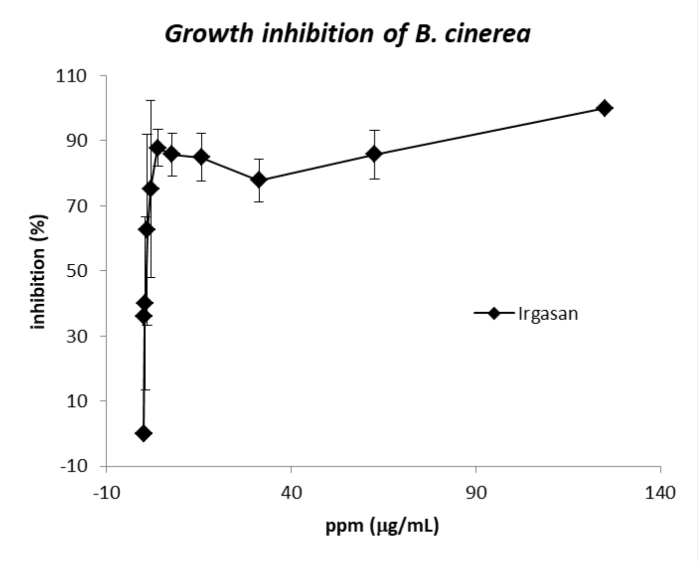

Conc. (ppm)	62,50	31,25	15,63	7,81	3,91	1,95	0,98	0,49	0,24	0,12	0,06
**Inhibition (%)**	85,69	77,88	84,89	85,73	87,74	75,27	62,52	39,93	35,89	0,00	0,00
**Standard deviation**	7,55	6,58	7,45	6,51	5,62	27,20	29,32	26,66	16,40	0,00	0,00

Log EC_{50}= -0.6360

EC_{50} = 0.2312 ppm

EC_{50} lower 95% confidence limit = 0.1862 ppm

EC_{50} upper 95% confidence limit = 0.2871 ppm

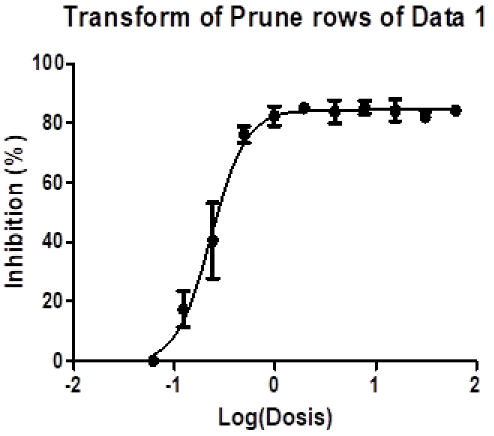

Error estandard LogEC50= 0.04596
(debe ser <0.5; EC50 valido)
R^2=0.9454

SI- 31- Positive control. Plate Bioassay (irgasan).

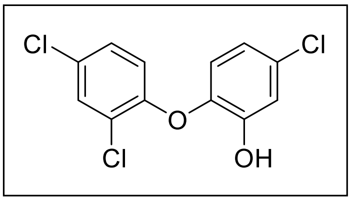

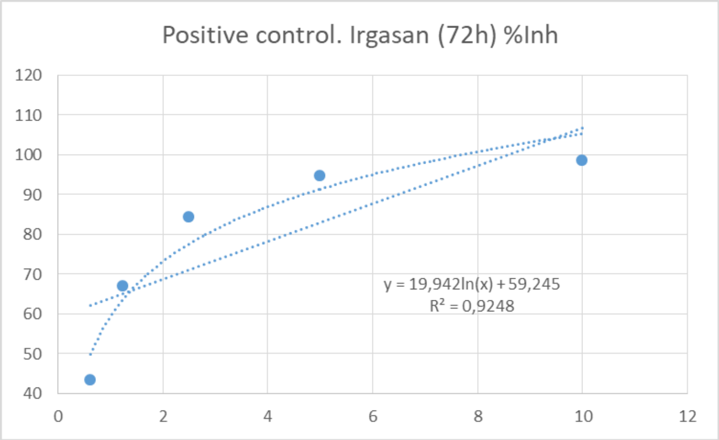

**IC50 (ppm)**	0.63
Rango EC50 inferior (ppm)	-
Rango EC50 superior (ppm)	-
**IC50 (µM)**	2.19
Rango EC50 inferior (µM)	-
Rango EC50 superior (µM)	-

**Irgasan (72h)**	
**Conc**	**%Inh**
10	98,53
5	94,57
2,5	84,32
1,25	66,94
0,625	43,23
